# Evaluating sustainability scenarios in coastal zones of Small Island Developing States (SIDS): A case study of the Dominican Republic

**DOI:** 10.1007/s13280-026-02345-7

**Published:** 2026-02-19

**Authors:** Natividad Pantaleón, Abel E. Camilo, Marina Rodes Blanco, Miguel A. Zavala, Antonio Gómez Sal

**Affiliations:** 1 Grupo de Ecología y Restauración Forestal (FORECO), Departamento de Ciencias de la Vida, Universidad de Alcalá, 28805 Alcalá de Henares, Madrid Spain; 2 Facultad de Ingeniería y Arquitectura, Universidad Autónoma de Santo Domingo (UASD), 10103 Santo Domingo, Dominican Republic; 3 Grupo de Ecología y Restauración Forestal (FORECO) and Global Change Ecology and Evolution (Glocee), Departamento de Ciencias de la Vida, Universidad de Alcalá, 28805 Alcalá de Henares, Madrid Spain

**Keywords:** Development scenarios, Land use, Multi-criteria analysis, Territory and sustainable planning

## Abstract

Assessing sustainability in coastal zones, especially in Small Island Developing States (SIDS) like the Dominican Republic, requires a multidimensional approach due to ecological fragility and social complexity. This study evaluates sustainability across 17 coastal provinces, home to 69% of the national population, using five dimensions: ecological, economic, productive, cultural, and social. A total of 148 indicators were selected to reflect key development drivers. Each dimension was independently assessed using Detrended Correspondence Analysis (DCA), followed by an integrated comparison through Discriminant Analysis against theoretical development scenarios. Results show that 75% of provinces face intense pressure on natural resources, while 25% exhibit more diversified and sustainable practices, though planning gaps persist. The approach enables identifying sustainability deficits and development trajectories, providing a replicable, context-sensitive tool for decision-making at multiple levels. Beyond the Dominican Republic, the framework can support coastal regions worldwide in pursuing resilient, inclusive, sustainable development pathways and informing evidence-based planning.

**Supplementary Information:**

The online version contains supplementary material available at 10.1007/s13280-026-02345-7.

## Introduction

The twenty-first century presents significant challenges in managing human activities and their impacts on coastal systems, where 5% of habitable land hosts 40% of the world’s population (MEA 2005). As ecological transition zones, coastal areas play a critical role in linking terrestrial and marine ecosystems. These regions support diverse habitats essential for human settlement, economic activities, and the livelihoods of local communities (Steven et al. 2020). However, increasing pressures threaten vital systems, particularly through biodiversity loss, pollution, and climate change impacts (IPBES 2019).

Globally, around 600 million people rely on fishing and aquaculture for their livelihoods. In the Caribbean, these sectors contribute significantly to food security, poverty reduction, and socioeconomic stability (FAO 2024). Coral reefs alone generate an estimated USD 11.5 billion annually through tourism (Masson-Delmotte et al. 2019), highlighting the economic importance of healthy coastal ecosystems.

Despite their value, oceans and coastal areas are highly vulnerable, undergoing rapid environmental changes that reduce their resilience (MEA 2005). Nearly 50% of brackish marshes (Mcowen et al. 2017), 35% of mangroves (Bunting et al. 2018), and 30% of coral reefs have been lost globally (Rogers and Miller 2016), accelerating the need for effective coastal management (Cicin-Sain and Belfiore 2005; UNEP 2020).

Sustainability in these territories requires balancing ecosystem preservation with social and economic adaptation (Adger 2000; Gallopín et al 2001). This balance is critical for Small Island Developing States (SIDS) like the Dominican Republic. Geographic isolation, limited resources, and increasing external pressures amplify vulnerabilities (Duvat et al. 2019). With 69% of its population living in coastal areas, the Dominican Republic faces compounded challenges from climate change, resource overuse, and rapid urbanization (Ministerio Ambiente 2012).

To address these complexities, sustainability assessments must be context-specific, integrating ecological, productive, socioeconomic, cultural, and governance aspects (Sala et al. 2012). Transdisciplinary approaches strengthen decision-making by linking local realities to global sustainability goals (Rockström, et al. 2009; St Flour and Bokhoree 2021; Fanning et al. 2022). Integrated and participatory methodologies foster interdisciplinary collaboration, address cultural and ethical elements, and utilize spatial analysis to support informed territorial planning (Geneletti and Ferretti 2015; Portman 2016).

In coastal territories, a multidimensional perspective is essential to understand nature–society dynamics for sustainable development (Ness et al. 2007). Tools such as threshold models, indicators, multi-criteria analysis, composite indices (Waas, et al. 2014; Boggia et al. 2017; Molina et al. 2022), and geospatial data, enable comprehensive assessments of environmental, socio and economic interactions (Cinelli et al. 2014).

A major challenge is developing models that reflect the complexity of sustainability dimensions (Gómez Sal et al. 2003). Selecting robust socio-ecological indicators is crucial to effectively capture these dynamics and informing territorial strategies (Holmberg and Karlsson 1992; Müller et al. 2000). Refining these indicators improves the analysis, even though they involve certain interpretative challenges (Azar et al. 1996; Ramos 2019).

Furthermore, multidimensional models combined with multivariate analysis enable effective indicator selection and tailored approaches, as long as dimensions maintain independent objectives despite functional connections (Gómez Sal 2001). Scenario-based approaches help address uncertainties, offering pathways for resilient development (Rosenbaum et al. 2012; Fauré et al. 2017). These methodological advances are particularly relevant for territorial planning in coastal zones, where ecological, social, economic, and cultural dynamics intersect (Gallopín et al. 2001; Folch 2003; St Flour and Bokhoree 2021).

In SIDS, coastal planning plays a pivotal role in mitigating vulnerabilities and aligning development with conservation priorities (Portman 2016). Achieving this requires not only technical tools, but also inclusive governance and institutional coordination (Burby et al. 2000; ECLAC 2019).

Despite progress in sustainability frameworks, such as Nature-Based Solutions (NbS) (Cohen-Shacham et al. 2016), Ecosystem-Based Adaptation (EbA) (CBD 2009), the Circular Economy (Geissdoerfer et al. 2017), Integrated Coastal Zone Management (ICZM) (Cicin-Sain and Belfiore 2005), the Blue Economy (Eikeset 2018), and participatory governance models (Wiek 2012), the systematic use of scenario-based approaches to guide decision-making and planning remains limited (Duvat et al. 2019). As a result, many interventions still lack the anticipatory perspective needed to confront future uncertainties, particularly in coastal SIDS (IPBES 2019; IPCC 2022).

This study evaluates the sustainability of the Dominican Republic’s 17 coastal provinces using a structured multi-criteria analysis. Conceptualizing sustainability as a multidimensional process, the analysis compares outcomes across theoretical development scenarios identifying trade-offs and synergies throughout territories. By defining and quantifying these dynamics, the study supports informed territorial planning and reveals critical socio-environmental interconnections (Díaz-Duque and Gómez Sal 2013; Geneletti and Ferretti 2015).

Aligning territorial sustainability assessment with global agendas like the Sustainable Development Goals (SDGs) is essential, particularly in SIDS. The methodological approach adopted in this study, combining multidimensional assessment with cluster analysis, provides a replicable framework for understanding territorial dynamics, informing differentiated development strategies, and guiding adaptive territorial planning aligned with both local realities and global priorities (Mendoza and Prabhu 2000; Gallopín et al. 2001; Munda 2004). This contributes not only to diagnosing the sustainability status of coastal provinces, but also to advancing integrated, inclusive, and resilient development pathways.

## Materials and methods

### Study area

**Fig. 1:**
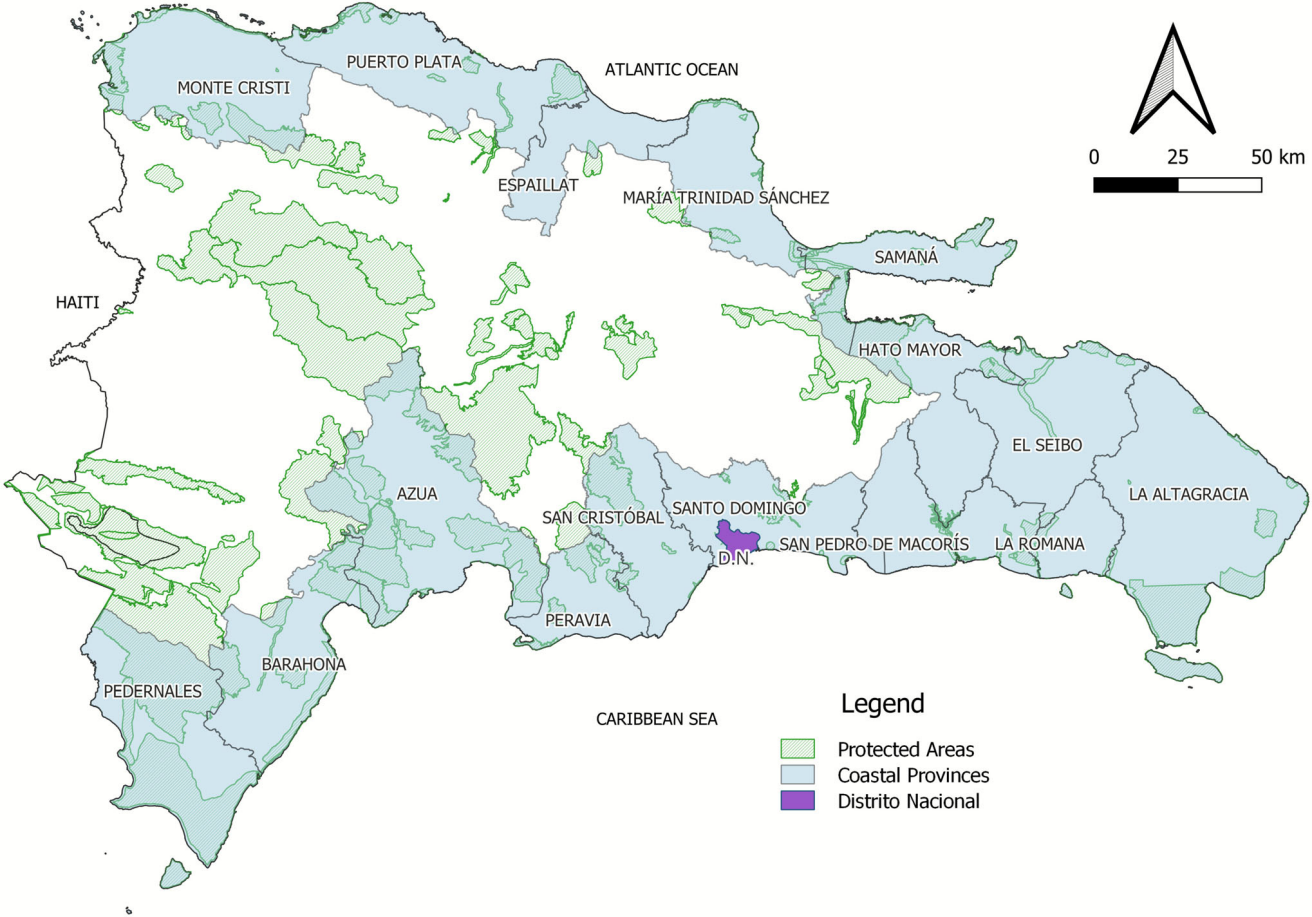
Dominican Republic coastal provinces and their protected areas

The study area encompasses the 17 coastal provinces of the Dominican Republic (DR), including its capital, Santo Domingo, National District (Fig. 1). These provinces represent 69% of the country’s population (ONE 2022), residing in diverse settings ranging from large urban centers to low-density rural communities. Economic activities vary widely, including subsistence agriculture, artisanal fishing, large-scale farming, industrial production, small businesses, mass tourism, and mining.

Climatic variations range from semi-arid to very humid. The average annual temperature is 25 °C, with small seasonal variations; average annual rainfall varies drastically from 455 mm southwest provinces to 2743 mm along the northeast coast (Ministerio Ambiente 2012). According to the Holdridge classification (OAS 1967), nine life zones and seven transitional formations were defined in the country, the Subtropical Moist Forest occupies the largest extension; followed by the Subtropical Dry Forest.

The coastal profile (1668.3 km) is characterized by being irregular, with steep reefs and swampy lands forming diverse landscapes: 41 locations with rocky coasts, 21 dune areas, 141 coastal lagoons, 181 reef areas, over 55 mangroves areas, 49 estuaries and 226 beaches. The National System of Protected Areas (NSPA) has 127 conservation units equivalent to 25.07% of the country (Fig. 1), under the categories based on the International Union for Conservation of Nature (IUCN) principles; 27 are marine protected areas (Ministerio Ambiente 2012).

### Variable selection

For this study, we considered five development dimensions: Ecological, Economic, Productive, Cultural and Social. All dimensions were evaluated through a set of selected variables (Table S1). The ecological and cultural variables were obtained, mainly, from the Atlas of Biodiversity and Natural Resources of the Dominican Republic (Ministerio Ambiente 2012), Recognition and Evaluation of Natural Resources in the Dominican Republic (OAS 1967), and the Uses of Biological Resources and Traditional Knowledge (Ministerio Ambiente 2018b). The socioeconomic and productive data from the National Population and Housing Census (ONE 2022), the Provincial Profiles and Provincial Development Plans (MEPyD 2017); among others (Matrix S1).

We carefully selected variables to ensure a comprehensive assessment of integrated sustainable development within the territory, focusing on its capacity for sustainability, integrity, and functioning. This includes evaluating its ability to maintain essential ecological processes, environmental services, and conservation value (ecological dimension); the infrastructure, technology, management systems, techniques, and natural resource use that support the provision of goods and services (productive dimension); economic activities such as agriculture, tourism, exports, and mining that sustain livelihoods (economic dimension); and preservation and valorization of ancestral heritage, traditional knowledge, and modern scientific understanding, along with the capacity to adapt to new challenges and apply empirical and rural cultural knowledge (cultural dimension).

Additionally, we consider aspects related to education, social cohesion, solidarity, identity, equitable wealth distribution, the provision of socially valued services, and the maintenance of stable demographics (social dimension). Each dimension was evaluated independently through multivariate analysis, which enabled the integration of multiple variable contributions. The five dimensions were defined based on key sustainability drivers in coastal SIDS, but the framework remains adaptable to different territorial contexts.

### Land use patterns and productive activities

Using the variables of the five dimensions, we performed an exploratory hierarchical classification analysis (Ward’s statistics) (Ward Jr. 1963), obtaining homogeneous linear segments of coastal provinces with similar land use patterns and productive activities (consolidated clusters). We called these Development Coastal Areas (DEPCA). The provinces clustering was made with the statistical software PAST 4.03 (Hammer et al. 2001).

### Data analysis

We developed an integrated, multi-step analytical approach to assess the sustainability performance of the 17 coastal provinces (analysis units) of the Dominican Republic (Hill & Gauch 1980). First, we calculated quantitative scores for each province across the five sustainability dimensions, based on a selection of relevant indicators (Table S2). To ensure comparability across variables with different units of measurement, the values were scaled between 0 and 100. To explore the data structure and better understand the relationships between variables within each dimension, we applied multivariate ordination techniques. Specially, we used Detrended Correspondence Analysis (DCA) to obtain standardized outputs across dimensions (Table S3). Following this, we performed a correlation analysis, using PAST 4.03 (Hammer et al. 2001), to determine the contribution of individual indicators to the overall dimension scores.

**Fig. 2:**
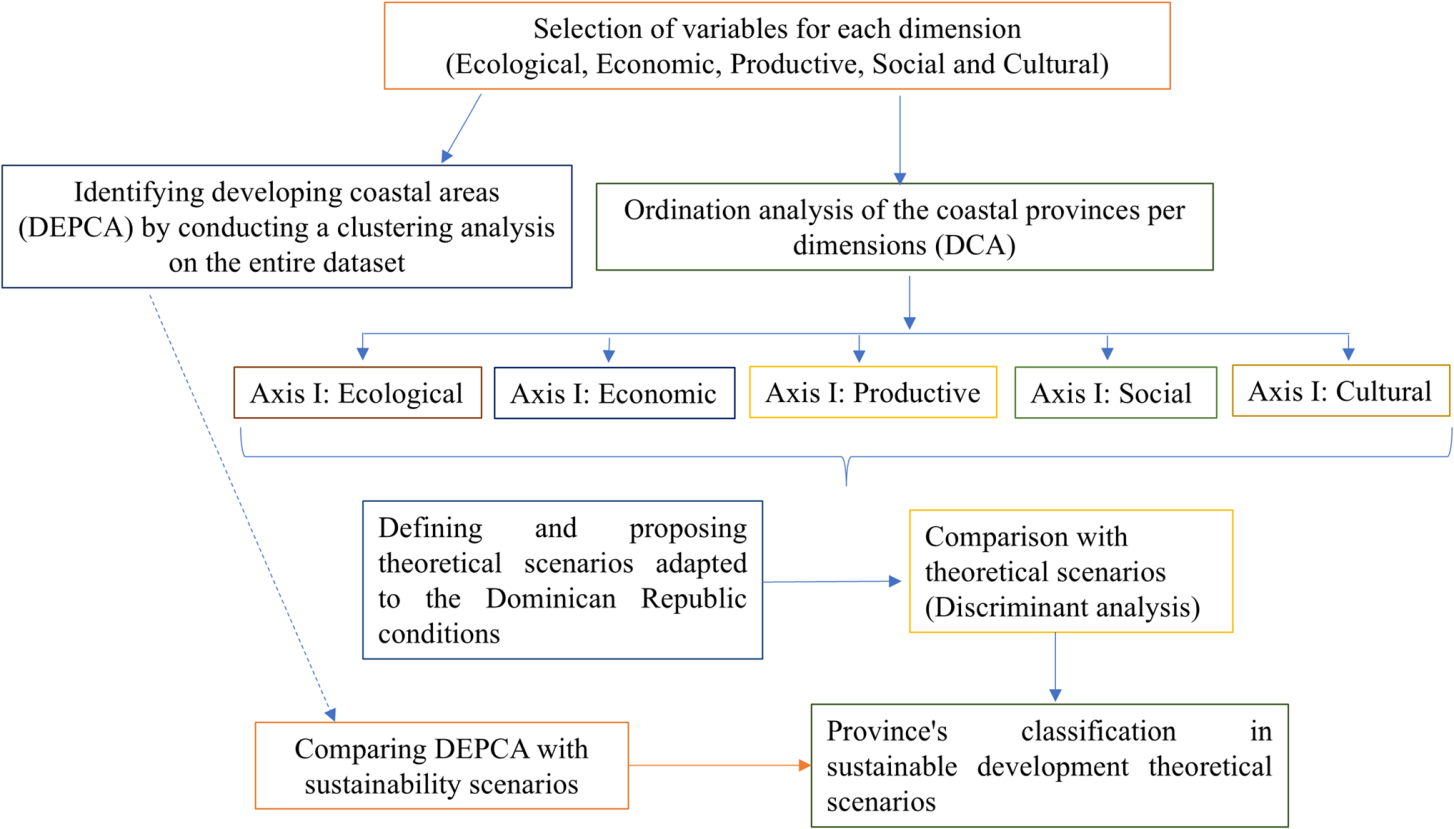
Description of the different stages of the multidimensional sustainability analysis applied to the Dominican Republic coastal provinces

This process resulted in a coherent and comparable set of sustainability value for each province, providing the basis for the integrated assessment and classification outlined in the next section. The steps of this multidimensional sustainability analysis are illustrated in Fig. 2.

### Integration of dimensions and comparison with theoretical development scenarios

**Fig. 3:**
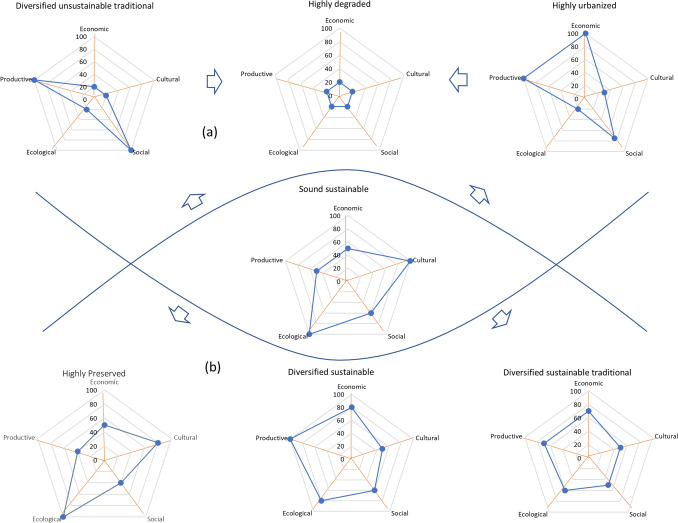
Theoretical sustainability scenarios based on five dimensions (ecological, social, economic, cultural, and governance), adapted to the coastal provinces of the Dominican Republic. Panel **a** represents trajectories associated with ecosystem degradation; panel **b** includes scenarios oriented toward the conservation of ecosystems and their services. Scenarios are intended as analytical tools to explore development pathways, not as normative or fixed outcomes

After calculating the sustainability scores for each dimension, we integrated these results to perform a comprehensive, multidimensional assessment of the coastal provinces. This enabled comparison of their overall sustainability performance against predefined theoretical development scenarios (Fig. 3, Table 1). To ensure an objective classification and accurately assign provinces to their most representative scenario, we applied Discriminant Analysis (DA) using PAST 4.03 (Hammer et al. 2001). This approach revealed predominant development patterns and provided insights to support sustainable planning. The five dimensions were selected for this case to reflect key sustainability aspects in coastal SIDS, especially the role of governance, but the framework remains adaptable to other contexts.

**Table 1:** Main characteristics of the theoretical development scenarios considered for the evaluation, adapted from those proposed by Gómez Sal (2001) and Gómez Sal and González García (2007)

Theoretical scenarios	Description
Highly preserved	High ecological and cultural value. Strong emphasis on conservation. Socioeconomic development remains limited, possibly due to structural or planning limitations
Sound sustainable	Rational utilization that preserves ecosystem processes, increasing sustaining capacity and natural capital. Economic value is improved by elevating the quality of products (sustainable tourism, ecological agriculture, etc.)
Diversified sustainable	Diversified productive activities with low impact on ecosystems. High levels of natural resources protection that preserve the ecosystem processes
Diversified sustainable traditional	Production based on traditional activities with a high appreciation of nature. Sustainability demands attention and effort
Diversified unsustainable traditional	Production based on traditional activities with a high impact on natural and cultural resources. Loss of ancestral knowledge and biodiversity
Highly urbanized	Highly urbanized. Production based on commercial, industrial, and service activities. Threatened and degraded ecosystems
Highly degraded	Low productivity due to soil degradation, polluted and scarce water, high loss of biodiversity, ecosystems, and cultural resources

To validate the independence of each dimension and prevent redundancy, we conducted Pearson’s correlation analysis, which verified that each dimension contributed uniquely to the integrated assessment. All statistical procedures, including correlation tests and Discriminant Analysis, were conducted in PAST 4.03, while data preprocessing and initial ordination were carried out using the vegan package (Oksanen et al. 2022) in R 4.2.1 (R Core Team 2022).

## Results

### Land use patterns in DR coastal provinces

**Fig. 4:**
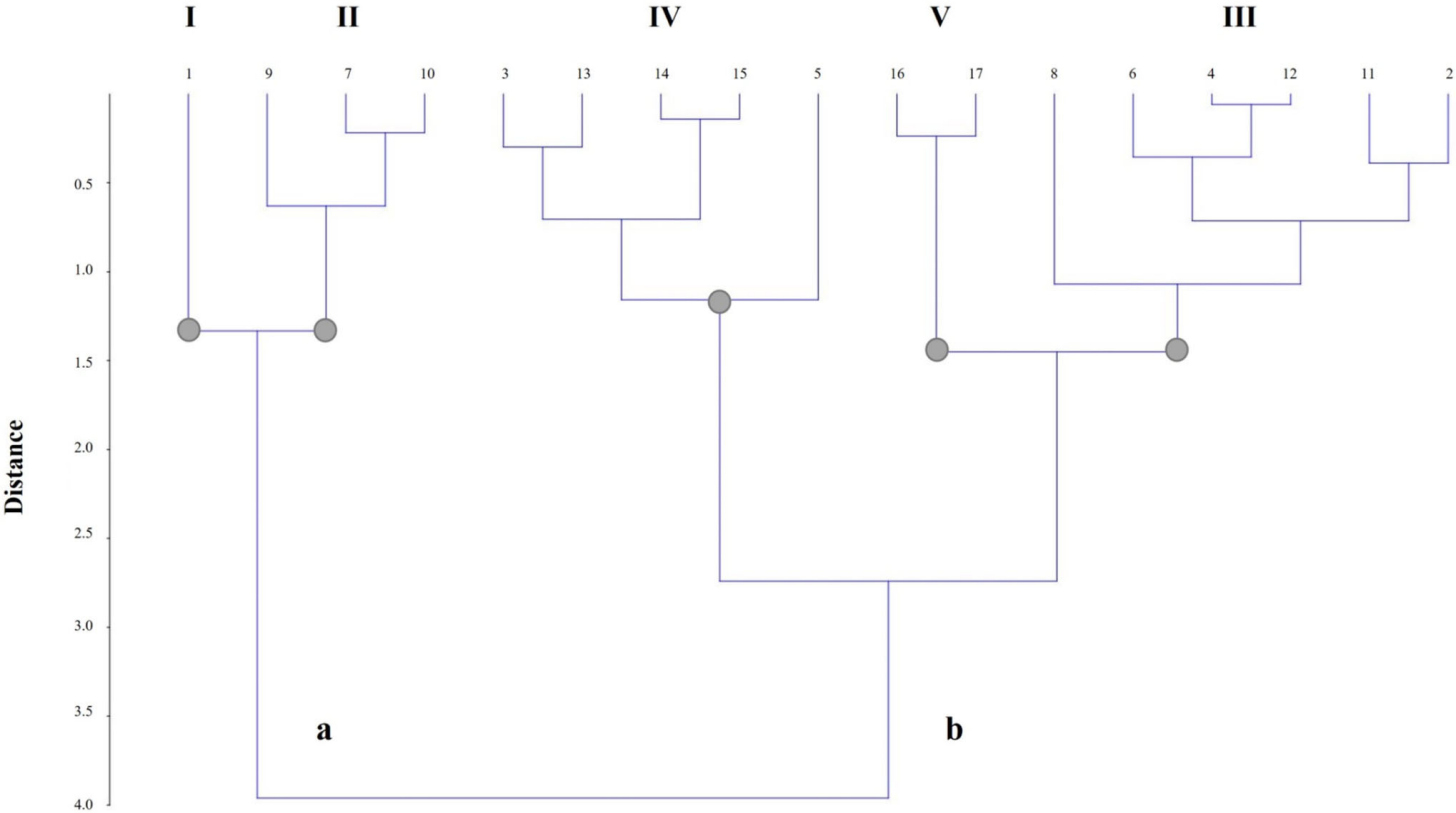
Classification of 17 provinces into five sets of units of occupation and use of the territory. **A** Comprise the 4 provinces with the highest private sector investment index and high presence of infrastructures; and **B** provinces (13) with greater heterogeneity in the land use, variety of life zones, endemism and conservation areas

Using variables from the five analytical dimensions, we conducted an exploratory hierarchical cluster analysis (Ward’s method) to identify homogeneous groups of coastal provinces sharing similar land use patterns and productive activities. The analysis identified two primary territorial clusters, further divided into five detailed subgroups (Fig. 4).

The first cluster, comprising 4 provinces, includes areas characterized by higher levels of private investment and infrastructure development, predominantly associated with tourism and real estate. The Subtropical Moist Forest life zone predominates in these provinces.

The second, larger cluster includes 13 provinces with more heterogeneous territorial structures. These areas feature a broader mix of land uses and productive sectors, including agriculture, livestock, artisanal fishing, mining, and commerce. They span multiple life zones and generally encompass a higher proportion of protected areas, with greater ecological diversity and conservation significance.

This classification reveals distinct contrasts in land use strategies and development intensity across the coastal provinces, offering a spatial foundation for differentiated sustainability assessments and targeted territorial planning. Although not part of the formal evaluation procedure, this initial joint analysis provided useful insights into territorial challenges and the relative importance of key variables, which informed the later selection of analytical dimensions, such as governance, and the design of plausible development scenarios.

Table 2 provides an overview of the Development Coastal Areas (DEPCA) categories and their corresponding provinces. The characteristics of each DEPCA were established based on predominant land uses and patterns of territorial occupation. For every DEPCA category, we identified a canonical unit, the province whose indicators are closest to the average values of its respective group (Fig. S1).

**Table 2:** Land use categories determined from the classification of the 17 provinces, using as variables the coordinates of axis 1 obtained from the DCA, including all variables

Category (DEPCA)	Description	Provinces
I. Highly urbanized—infrastructures—commerce	Highly urbanized with Subtropical Moist Forest patches, mostly covered by infrastructure (houses, highways, industries, hotels, etc.). Limited artisanal fishermen, rocky coastline with river mouths, few dunes/beaches, and numerous caves of archeological significance	1. National District
II. Private sector investment—tourism & infrastructures	Subtropical Moist Forest predominates. Coastline with numerous beaches. High private sector investments are mostly associated with tourism. Important tourist infrastructures. A large percentage of the territory is dedicated to agriculture, livestock, and artisanal fishing. High-medium scale tourist activity. Medium to high urban development, and low-density rural communities	9. El Seibo 7. Samaná 10. La Altagracia
III. Diversified productive—tourism—agriculture & livestock	Subtropical Moist Forest predominates. Coastline with numerous beaches and dunes. A high percentage of the territory is dedicated to agriculture and livestock. Artisanal fishing and medium scale tourism. Medium to high urban development, and rural communities with a low to medium density. Significant number of caves of archeological importance	8. Hato Mayor 6. María Trinidad Sánchez 4. Puerto Plata 12. San Pedro de Macorís 11. La Romana 2. Santo Domingo
IV. Agriculture—livestock, mining productive	More than half of the territory is occupied by Subtropical Moist Forest. Coastline with numerous beaches, dunes, and mangrove forests. Agricultural and livestock activities predominate. Artisanal fishing and small-scale tourism. Metals or non-metals mining. Important number of protected areas. Medium urban development, and low-density rural communities	3. Montecristi 13. San Cristóbal 14. Peravia 15. Azua 5. Espaillat
V. Diversified small scale productive—highly preserved	Abundant Subtropical Moist/Dry Forest with diverse life zones and high endemism. Coastal areas feature numerous beaches. The territory is mainly used for subsistence agriculture, livestock, and significant artisanal fishing. It has small-scale ecotourism, low infrastructure, medium urban development, and low-density communities. A large percentage is designated as protected areas, with a notable number of caves of archeological importance	16. Barahona 17. Pedernales

### Multidimensional sustainability in DR coastal provinces

The results for each dimension obtained of the DCA analysis and the variables that contribute the most to the definition of the first axis are described below.

#### Ecological dimension

In the ecological dimension, high values are observed in the provinces of Pedernales (100), Barahona (89.9) and Azua (81.6); followed by Peravia (71.0), and Montecristi (69.9). All with heterogeneous life zones, high endemism, and a large percentage of their territories as Protected Areas. In contrast, the National District (0.0) shows very low values with highly urbanized areas and limited natural spaces. Maria Trinidad Sánchez (48.0), Samaná (43.7) and La Romana (32.7) show low numbers *in forest* and *life zone types.* The rest of the provinces show values between 54.4 and 62.2 (Fig. S2).

The primary meaning of Axis I within the ecological dimension emphasizes the prevalence of variables such as high endemism, heterogeneous ecosystems and life zones, altitudinal range, and natural ecosystems that are predominantly protected or only slightly transformed. The variables with positive values of axis I (Table S4) are *endemic species* (0.96), *humid forests and transition forests* (0.94), *lakes and lagoons* (0.86), *subtropical dry forest* (0.64), *altitudinal range* (0.60), *heterogeneous life zones* (0.52) and *protected areas* (0.47). The variables *droughts threat* (0.53), *fires threat* (0.31), *landslides threat* (0.28) and *floods threat* (0.27), showed important values; however, the values for threats to *hurricanes* (0.17), *seismic* (0.17) and *vulnerability index* (0.17), are low.

Variables with very low values are coverage of *Subtropical Moist Forest* (0.9), *Subtropical Wet Forest* (0.7), *soils suitable for agriculture* (0.5) and *rainfall* (0.5).

#### Productive dimension

The high values are associated with the use of structures for the modernization and efficiency of agricultural production *(irrigation canals, production in controlled environments, paved access roads, renewable energy,* etc.), giving it a high significance to *agricultural production*, especially in Espaillat (100), Azua, (70.2), Peravia (69.7) and San Cristóbal (52.5); and medium relevance in Montecristi, (34.7), El Seibo (34.2) and Hato Mayor (32.2). In provinces with low modernization in agricultural production and limited access to irrigation canals, also show a low productivity level, e.g., Samaná (1.7), San Pedro de Macorís (1.2) and Puerto Plata (0.7) (Fig. S3). The Productive Dimension shows *agriculture and livestock production* as the most relevant activities; *artisanal fishing* and *subsistence agriculture* are also vital for coastal communities.

Axis I of the Productive Dimension is explained by the variables (Table S5) of the largest *number of controlled environment crops* (0.99), *access to water for agricultural production through irrigation channels* (0.74), *number of agricultural producers* (0.70), *subsistence agriculture* (0.67), *irrigated cultivation* (0.66), *use of wild flora and fauna* (0.52); and by the province infrastructure for production: *paved roads* (0.63), *renewable energy use* (0.62), *internet access* (0.51), *commercial docks* (0.50) and *artisanal landing ports* (0.49).

With very low values are the variables: *Visits to Protected Areas* (0.19), *number of hotels* (0.19), *tourist piers* (0.15), *hotel rooms* (0.2), *hotel beds* (− 0.3) and *tourism water demand in m*^*3*^ (− 0.4).

#### Economic dimension

The Economic Dimension is marked by highly profitable economic activities such as *tourist airports arrivals*, *private sector investments in construction*, and *volume of metallic and non-metallic minerals mined* in the province. In several provinces the percentage of the *Economically Active Population* predominates, a high number of *formal Micro, Small and Medium Enterprises* (MSMEs), and *employment in the tertiary sector*.

Samaná (100), El Seibo (98.6) and La Altagracia (96.8), show high values, as provinces with expanding tourism development, followed by the National District (56.2) which benefits from large private sector investments and employment in the service sector. Hato Mayor (50.1) registers a high position with significant levels of *Economically Active Population*, *private investment in the construction sector* and products of high added value, such as *metallic and non-metallic minerals extraction*. The remaining provinces maintain low levels, ranging between 0.0 and 13.9 (Fig. S4).

Axis I of the Economic Dimension (Table S6) is based on variables such as: *Airports foreign arrivals* (0.99), *private sector investments in construction* (0.83), *formal MSMEs* (0.75), *employees in the tertiary sector* (0.65), *Economically Active Population* (0.63), *number of mining concessions* (0.61), and *extraction of metallic and non-metallic minerals* (0.49). With very low values, the variables with the highest incidence are related to free zones: *free zones employees* (0.36), *free zones exports* (0.33), and *free zones investments* (0.15).

#### Cultural dimension

In the Cultural Dimension, the variables *women with university studies* and *women participation in local government positions*, predominate. The variables *cultural heritage and traditional knowledge adapted to the resources, empirical knowledge, rural culture, conservation and valorization of manifestations, rituals and folk dances, crafts in ancestral materials and techniques*, are also valued. The *number of caves with archeological-cultural importance places* Pedernales (100) at the top; followed by 14 more provinces with medium–high values between 45.0 and 84.6. The National District (0.0) and Santo Domingo (23.9) show low values in variables as *traditional cultural manifestations conservation and appreciation of ancestral techniques in the creation of craft products* (Fig. S5).

The primary meaning of axis I of the cultural dimension (Table S7) is *education*, *learning and ability to face new challenges*, as well as a *high appreciation for traditional manifestations and ancestral techniques in the creation of artisanal products*. The valorization of the variables: *women participation in local governments decisions, numbers of women in universities* and *women enrolled in secondary school* are high.

The variables with positive values of axis I are linked to adapted skills and knowledge about resources, and empirical and rural cultural knowledge: *women with university education* (0.99), *positions in local governments held by women* (0.99), *valued folk dances* (0.91), *preserved ritual and occasional folk dances* (0.89), *handicrafts in ancestral materials and techniques* (0.84). Other positive values are the relevance of natural and cultural heritage in the territory, such as *caves with archeological-cultural importance* (0.82), and the ability to face new challenges, such as *number of students enrolled in secondary school* (0.52). The variables *public use libraries* (0.41) *number of museums and cultural centers* (0.34) showed medium–low values.

#### Social dimension

In the Social Dimension, prevails the variables: *Population density, access to higher education and technical careers, number of universities* and *population enrolled in universities*. *Access to public health and physicians*’ *number (per 10 000 inhabitants)* show high values; placing the National District (100) in the highest place, separated significantly from the rest of the provinces. The values in the *number of people enrolled in universities* are high in Barahona (44.4), María Trinidad Sánchez (40.3), Peravia (29.4) and La Romana (29.1); however, they show much lower levels than those registered in the National District. High values are recognized in *homes with access to paved roads*, *population*’*s access to drinking water services, homes with access to landlines phone service,* and *the number of educational centers for basic, middle, and secondary levels*. The provinces with the lowest levels in the social dimension, Samaná (4.2), Pedernales (6.3), El Seibo (0.3), and Montecristi (0.0) show high results in *unemployment rates, immigration rates,* and *the proportion of households living in general poverty* (Fig. S6).

The Axis I of the Social Dimension (Table S8) is explained by the variables related to *population density* (0.99), *students enrolled in universities* (0.98); and other variables of higher education, such as: *Number of private universities* (0.87), *total of public or private universities* (0.85), and *population enrolled in technical-higher education* (0.67). Variables on access to public health with high values: *Number of public hospitals* (0.74), *average of doctors per 10 000 inhabitants* (0.72), and *public primary care health centers* (0.71). The variables related to public services with the highest valuations are *Households with access to landlines phones* (0.74), *households with paved access roads* (0.56), *population access to drinking water services* (0.53), *and number of educational centers* (0.49). The variable on the *provincial Human Development Index (HDI)* shows a value of 0.55.

The low values in the social dimension are mainly associated with human development. Among the variables with low valuation are: *Households with exposure to natural disaster risk* (0.33), *immigration rate* (0.30), *unemployment* (0.30), *adolescent pregnancy rate* (0.28), *households in general poverty* (0.23), *illiteracy* (0.12), *populations with less than 10 000 inhabitants* (0.15), *households in extreme multidimensional poverty* (0.11), *households without sanitary service* (0.07) and *households that use charcoal or firewood as fuel for cooking* (0.6).

### Co-linearity among evaluative dimensions

We observed a low correlation among the five dimensions, suggesting their independence (Table 3), which is considered an indicator of robustness (i.e., the evaluation aspects should exhibit weak correlations to be regarded as valid dimensions, Gómez Sal et al. 2003).

**Table 3:** Correlations matrix (Pearson) between the five evaluative dimensions of development

Dimension	Ecological	Economic	Productive	Social	Cultural
Ecological	1	− 0.34013	0.25623	− 0.5934	0.65937
		*P* = 0.181	*P* = 0.320	*P* = 0.012	*P* = 0.004
Economic		1	− 0.22862	0.023385	0.019299
			*P* = 0.377	*P* = 0.929	*P* = 0.941
Productive			1	− 0.26304	0.068833
				*P* = 0.307	*P* = 0.792
Social				1	− 0.66956
					*P* = 0.003
Cultural					1

There is a medium negative correlation between the ecological and social axes that can be explained by the variable population density and its impact on natural resources. While the negative correlation between the social and cultural axes is justified due to an increase in population density, mostly in urban areas, it generates loss of knowledge and ancestral practices of the population. On the other hand, an average positive correlation is identified between the ecological and cultural axes, where there exists a directly proportional correspondence between the percentage of the territory of the provinces dedicated to conservation and the state of conservation of the natural and cultural heritage in the territory. In general, these relationships support the assumption of independence of the dimensions of the proposed model in the studied case.

### Dimensions integration: development scenarios present in DR coastal provinces

**Fig. 5:**
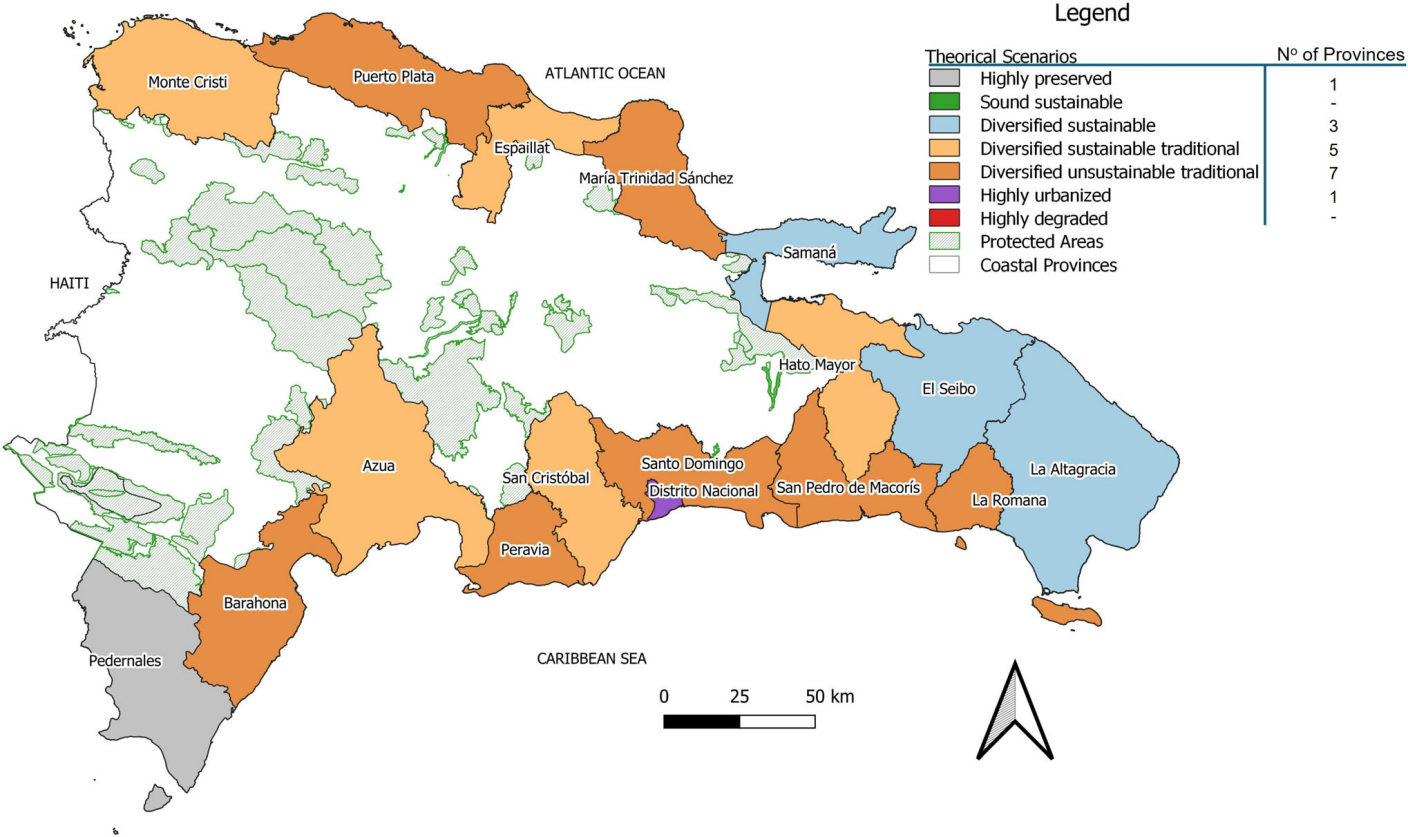
Map with the theoretical scenario’s models assigned to the 17 coastal provinces by the discriminant analysis

The Discriminant Analysis grouped 17 provinces into five of seven sustainability scenarios (Fig. 5). Over 75% belong to scenarios of high resource pressure and degradation, mainly *Diversified Unsustainable* and *Sustainable Traditional*, associated with coastal development, intensive agriculture, and livestock. The *Diversified Sustainable* scenario reflects a stronger appreciation for nature and ecosystem services. The *Highly Urbanized* scenario is exclusive to the National District, while the *Highly Preserved* scenario occurs in the province with the highest proportion of protected territory. *Highly Degraded* and *Sound Sustainable* scenarios were not observed.

The *Diversified Unsustainable Traditional* scenario encompasses seven of the 17 provinces. It is characterized by traditional productive activities with a high impact on natural and cultural resources, leading to biodiversity loss and erosion of ancestral knowledge. Agriculture and extensive livestock dominate, heavily impacting water sources and causing pollution. Low agricultural investment and high urban migration concentrate populations in cities, driving significant land-use changes and the expansion of poverty belts.

Five provinces fall under the *Diversified Sustainable Traditional* scenario, where traditional activities dominate alongside a strong appreciation for nature. Productive activities are more heterogeneous, with medium and subsistence-scale agriculture and livestock, complemented by service and commercial MSMEs. Montecristi stands out for its extensive monoculture crops, balanced by some of the country’s best-preserved mangrove forests and coral reefs, which cover approximately 36% of its territory.

Three provinces fit the *Diversified Sustainable* scenario, marked by diverse productive activities with low ecological impact and strong ecosystem protection. Tourism is a key driver, centered on beaches and protected areas. In Samaná, tourism focuses on real estate, whale watching, and beaches. La Altagracia features Bávaro-Punta Cana and Bayahibe, the Caribbean’s most visited destinations, dominated by mass tourism. El Seibo, in the early stages of mass tourism development, is attracting significant investment in tourist infrastructure.

The *Highly urbanized* scenario is present exclusively in the National District, the urban center with the highest population density in the country. It is characterized by having highly urbanized spaces, with production based mainly on commercial, industrial, and service activities. Its ecosystems are highly threatened and/or degraded, and aquifers and coastal-marine habitats highly polluted.

The *Highly Preserved scenario* represents territories with high ecological and cultural value, where a significant portion of land is designated as protected. In Pedernales, over 60% of the territory falls within protected areas, including critical zones of endemism inside the Jaragua-Bahoruco-Enriquillo UNESCO Biosphere Reserve. Despite these natural assets, the province experiences low levels of economic, productive, and social development, conditions largely driven by geographic isolation, weak infrastructure, and insufficient investment in inclusive development strategies. This scenario does not imply that conservation inherently restricts economic potential; rather, it illustrates how structural and institutional barriers can hinder development outcomes in ecologically rich areas.

## Discussion

### Territorial planning: balancing conservation and development

The sustainability scenarios identified in this study provide a structured lens through which to interpret the territorial development dynamics of the Dominican Republic’s coastal provinces. The coexistence of high ecological value and low socioeconomic performance in Pedernales (“*Highly Preserved*” scenario), and the inverse in the National District (“*Highly Urbanized*” scenario), underscores the fragmented nature of development in SIDS, revealing the absence of integrated territorial planning capable of reconciling environmental protection with inclusive economic growth.

These spatial and functional imbalances reflect structural deficiencies in land-use governance. Provinces such as La Altagracia and El Seibo, aligned with the “*Diversified Sustainable*” scenario, illustrate how tourism-led development, when guided by sustainability criteria, can generate economic value while safeguarding ecological assets, although they face significant pressures from rapid growth and environmental degradation. In contrast, provinces under the “*Unsustainable Traditional*” scenario, like Peravia, San Pedro de Macorís, and Barahona, show that persistence in traditional productive systems can exacerbate resource degradation without advancing socioeconomic well-being.

This reality highlights the limitations of sectoral planning models that overlook the complex interdependencies between ecological, social, cultural, and economic systems (Gallopín et al. 2001; Folch 2003). In coastal territories, shaped by diverse stakeholder interactions and local geographic contexts, planning must address multidimensional and evolving challenges (St Flour and Bokhoree 2021). This is particularly pressing in SIDS such as the Dominican Republic, where limited resources, institutional fragility, and climate vulnerability intensify territorial risks (Steven et al. 2020).

The weak correlations found among the sustainability dimensions reinforce the value of integrated assessment frameworks. Progress in one domain, such as economic growth, does not necessarily translate into advances in others, like ecological health or governance, supporting the need for integrated and adaptive territorial planning strategies. This aligns with calls for approaches that go beyond static land-use prescriptions to promote equity, resilience, and sufficiency in resource use (Fanning et al. 2022).

Embedding sustainability indicators into territorial decision-making is essential for aligning planning with the Sustainable Development Goals (SDGs). As demonstrated in this study, combining multidimensional evaluation with spatial clustering enables the design of territorial responses that are both context-sensitive and scalable. Advancing toward “*Sound Sustainable*” scenarios will require not only technical tools and diagnostic capacity, but also long-term institutional commitment, participatory governance, and multisectoral collaboration (Burby et al. 2000; ECLAC 2019).

### Land use patterns and sustainable challenges

Land use patterns are central to understanding sustainability dynamics in coastal zones, as they reflect both ecological pressures and socioeconomic strategies. In the Dominican Republic, these patterns are shaped by activities such as agriculture, tourism, and industrial development (Ministerio Ambiente 2018a). While these sectors support economic growth, they also contribute to environmental degradation, altering landscapes, fragmenting habitats, and accelerating biodiversity loss (Díaz et al. 2006). These transformations compromise ecosystem services that sustain human well-being and long-term resilience.

Territorial sustainability is thus closely linked to how land is used and managed. The relationship between economic production and environmental impact is mediated by land-use models, revealing the interdependence between ecological integrity and development strategies (Gómez Sal 2001). As shown in this study, provinces such as Pedernales and the National District illustrate contrasting dynamics: while Pedernales preserves valuable ecological assets yet remains economically marginalized, the National District undergoes strong economic growth alongside marked ecological degradation. These cases reflect fragmented land-use governance and underscore the need for more integrated and balanced territorial management.

The tension between conservation and productive use is a recurrent theme in coastal development. Gómez Sal (2009) argues that generic conservation models often fail in diverse local contexts, where place-based strategies are essential to address specific ecological and socioeconomic conditions. In Pedernales, where over 60% of the territory is under formal protection yet development indicators remain low, efforts should prioritize improving management and socio-ecological effectiveness rather than expanding protected areas.

This is particularly relevant in countries with weak institutional capacity and limited enforcement mechanisms (Fürst 2008). Our analysis reinforces these concerns. Provinces under the “*Unsustainable Traditional*” scenario, such as San Pedro de Macorís, María Trinidad Sánchez, and Peravia, remain dependent on conventional agriculture and livestock systems that degrade ecosystems without fostering inclusive development. These provinces exhibit low ecological value and only moderate productivity, revealing the constraints of unsustainable land-use models in the face of environmental stress and demographic pressure.

In contrast, provinces aligned with the “*Diversified Sustainable*” scenario, like La Altagracia and Samaná, demonstrate how adaptive land-use strategies can reconcile ecological conservation with economic opportunity. Tourism-led development, when regulated and supported by local institutions, enables income generation without compromising critical ecosystems. However, these provinces show signs of unplanned growth, which could threaten their current balance if not addressed through proactive governance and monitoring. This underscores the need for continuous oversight and anticipatory planning to safeguard their sustainability trajectories. Together, these findings affirm the importance of tailoring planning to each province’s context, recognizing the diversity of development pathways and risks. Ultimately, land use planning plays a decisive role in shaping sustainability outcomes. When informed by multidimensional assessments and local realities, it becomes a powerful tool for advancing resilient and inclusive coastal development.

### Development Coastal Areas (DEPCA): Territorial clustering, sustainability scenarios, and policy implications

The cluster analysis conducted in this study provides critical insights into the territorial development patterns of the Dominican Republic’s coastal provinces. By grouping provinces into Development Coastal Areas (DEPCA), the analysis reveals shared land use dynamics, ecological pressures, and socioeconomic characteristics, helping to explain how historical land occupation and development trajectories have shaped current sustainability challenges (Ministerio Ambiente 2018a).

This classification underscores the interdependence between ecosystems and societies in territorial planning, an aspect often overlooked by traditional approaches (Zambrano 2014). Such an integrated perspective aligns with global calls for holistic territorial management, particularly in SIDS, where ecological vulnerability and socioeconomic fragility often coincide (Bartley et al. 2001). The use of hierarchical clustering based on Ward’s method (Ward Jr. 1963; Everitt et al. 2001) proved effective in handling complex and dispersed datasets, enabling coherent groupings despite blurred boundaries and overlapping characteristics (González-Quiroz et al. 2021).

**Fig. 6:**
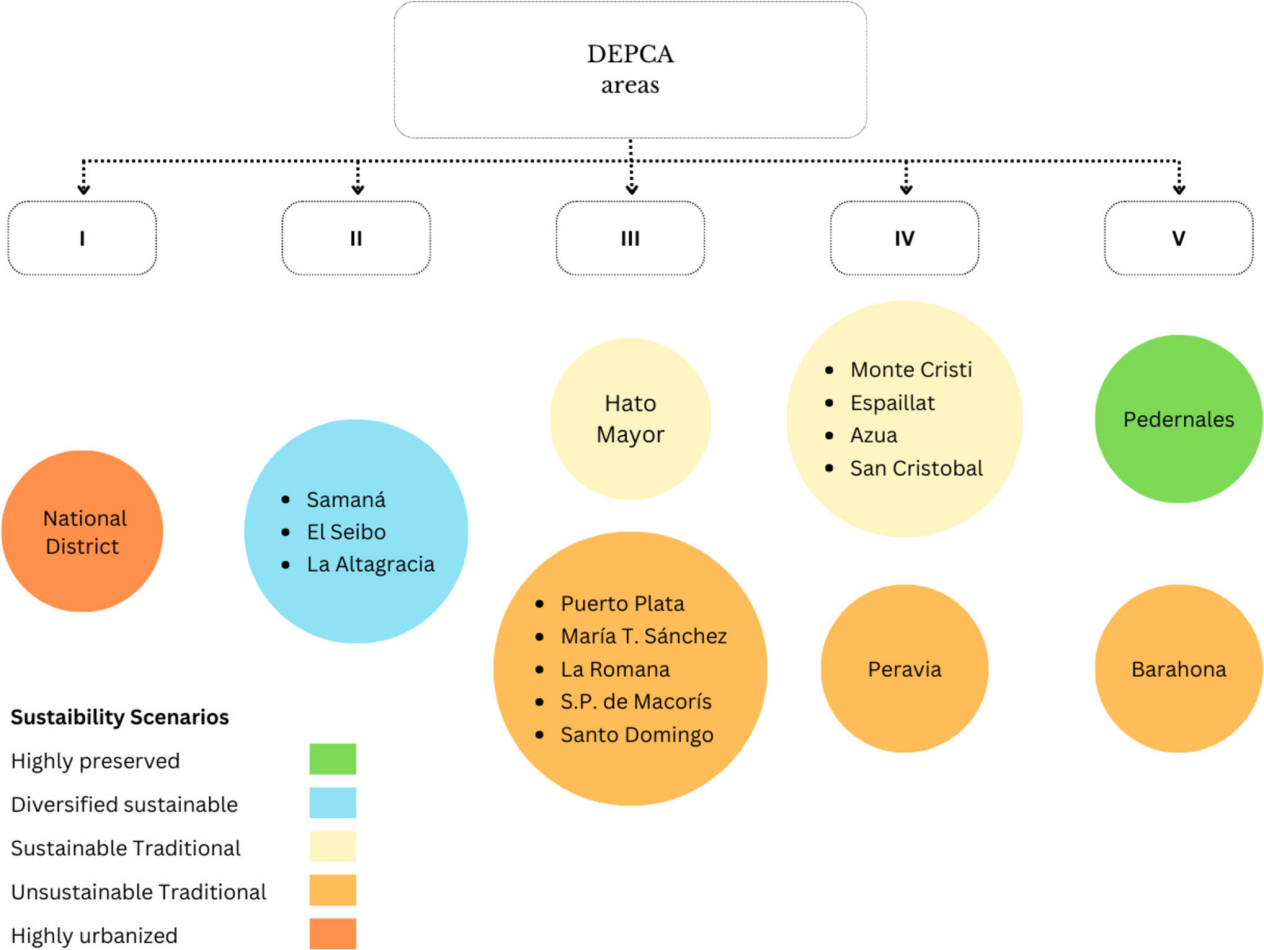
Contrasting Cluster Analysis results (DEPCA Zones) with multi-dimensional evaluation of actual sustainability scenarios

Importantly, beyond its statistical value, the clustering approach enhances the strategic dimension of sustainability planning. It enables the identification of provinces that require targeted interventions, such as regulated tourism development in ecologically sensitive areas, or ecosystem restoration in zones impacted by intensive agricultural. The observed alignment between DEPCA categories and sustainability scenarios reinforces the method’s relevance, offering clear patterns to inform policy and planning priorities (Fig. 6).

For instance, the National District (DEPCA I) corresponds with the “*Highly Urbanized*” scenario, highlighting urgent needs for infrastructure reform and ecological restoration. Pedernales (DEPCA V), meanwhile, aligns with the “*Highly Preserved*” scenario, illustrating trade-offs between conservation goals and socioeconomic development. DEPCA II provinces, like Samaná and La Altagracia, exemplify the potential of regulated tourism to balance economic growth and environmental integrity, whereas DEPCA III and IV provinces reflect ongoing tensions between traditional practices and sustainable land use.

Ultimately, the convergence between land use typologies and sustainability performance underscores the effectiveness of combining cluster analysis with multidimensional evaluation frameworks. This dual approach strengthens context-sensitive planning, enabling interventions tailored to the specific land use patterns, socio-ecological constraints, and development potential of each province.

### Integrating multidimensional sustainability assessment: insights for SIDS coastal management

Multidimensional sustainability analysis is essential for understanding the complex interactions among ecological, economic, productive, cultural, and social systems in SIDS. In the case of the Dominican Republic’s coastal provinces, the integration of cluster analysis with dimensional scoring revealed territorial configurations that move beyond sectoral boundaries, capturing interlinked patterns of land use, resource pressure, and institutional presence (Gómez Sal et al. 2003).

This analytical approach helped identify both synergies and tensions among sustainability components. Positive correlations, such as those between ecological and cultural values in provinces like Barahona and Montecristi, suggest potential for integrating biodiversity conservation with cultural heritage strategies. Conversely, negative correlations, e.g., between ecological and social conditions in the National District, highlight urban pressures that require better land-use and infrastructure planning.

The findings show divergent territorial trajectories. Provinces like Montecristi, Espaillat, Azua, and San Cristóbal correspond to the “*Traditional Sustainable*” scenario, where productive agriculture coexists with conservation values. In contrast, provinces such as San Pedro de Macorís, María Trinidad Sánchez, Peravia, and Puerto Plata, under the “*Unsustainable Traditional*” scenario, suffer environmental degradation linked to poorly regulated agricultural and livestock practices, with limited social returns. This demonstrates that sustainability is not solely determined by geography or economic activity, but also by institutional performance and land governance.

These differentiated outcomes emphasize the importance of aligning ecological and productive practices with policy capacity. As Falconi and Burbano (2004) argue, sustainability transitions require more than technical adjustments, they demand coherent policies, institutional coordination, and long-term stakeholder engagement.

### Advancing sustainability pathways and aligning with the SDGs

This study reinforces the relevance of applying multidimensional sustainability assessments to inform territorial planning in coastal SIDS, where ecological vulnerability, socioeconomic inequality, and institutional fragility converge. By combining cluster analysis with a context-sensitive evaluation framework, the research offers a practical tool for guiding adaptive planning, prioritizing strategic investments, and supporting evidence-based policy decisions.

The methodological configuration enables the identification of both shared patterns and province-specific challenges. By capturing local conditions and performance across multiple dimensions, it enhances the precision, equity, and effectiveness of policy interventions. As Gallopín (2001) highlights, regionally differentiated assessments are essential to understand dynamic systems and design appropriate responses. Similarly, Munda (2004) emphasizes the role of multidimensional evaluations in tailoring public policies to diverse territorial contexts.

The contrasting trajectories of provinces such as Pedernales and La Altagracia underscore the value of place-based strategies. Pedernales, with over 60% of its territory under protection, remains economically marginalized, partly due to persistent institutional and planning limitations. In contrast, La Altagracia demonstrates that, when supported by relatively stronger governance, tourism-led development can contribute to both economic growth and ecosystem protection, though significant challenges remain. These cases illustrate that integrating conservation and development is possible, but only when grounded in coherent, participatory, and adaptive strategies.

The absence of any province in the “*Sound Sustainable*” scenario highlights the need to overcome structural barriers such as fragmented institutions, limited cross-sectoral coordination, and underinvestment in inclusive development and environmental stewardship. Addressing these challenges will require sustained, multidimensional planning aligned with both national priorities and the SDGs. The deliberate selection of five interrelated dimensions, ecological, productive, economic, cultural, and social, proved particularly effective in this SIDS context, as it captured key territorial dynamics and institutional constraints. While the framework is adaptable and can be reconfigured for other settings, its structure in this study enabled a meaningful and context-sensitive understanding of sustainability conditions across Dominican coastal provinces.

As emphasized by Mendoza and Prabhu (2000), and Boggia et al. (2017), multidimensional frameworks are essential for operationalizing sustainable development, as they reveal synergies and trade-offs across ecological, social, and economic spheres. Without integrated planning, coastal provinces in the Dominican Republic will remain vulnerable to climate change, rapid urbanization, and biodiversity loss.

## Conclusions

This study provides a multidimensional assessment of sustainability across the Dominican Republic’s 17 coastal provinces, revealing significant variations in ecological value, land use patterns, and governance capacity. Over 75% of the provinces face substantial resource pressure, aligning with unsustainable trajectories such as the “*Unsustainable Traditional*” and “*Diversified Unsustainable*” scenarios. In contrast, provinces within the “*Diversified Sustainable*” scenario demonstrate that reconciling economic growth with environmental protection is feasible when supported by adequate planning and regulation. Nevertheless, even these provinces exhibit risks of unplanned growth, underscoring the necessity for ongoing monitoring and anticipatory governance.

By integrating a five-dimensional sustainability framework with hierarchical clustering, this study delivers a practical tool for policymakers to identify territorial patterns, similarities, and divergences across provinces. This approach enhances the accuracy and contextual relevance of interventions, particularly in Small Island Developing States (SIDS), where ecological fragility and resource constraints demand adaptive strategies.

The strength of this approach lies in its integration of multidimensional evaluation, contextualized through territorial clustering and compared against predefined scenarios. This methodological design reinforces the link between quantitative analysis and policy application, offering a replicable model for sustainability assessments in coastal and island contexts. The findings highlight the critical need for cross-sectoral coordination, inclusive governance, and long-term institutional commitment to guide coastal territories toward resilient and equitable development pathways.

Beyond its national relevance, the approach developed in this study provides strategic guidance for building resilient and inclusive coastal communities worldwide. As a scalable and adaptable framework, it offers practical tools for advancing coastal sustainability, aligned with the SDGs and the priorities of SIDS.

## Supplementary Information

Below is the link to the electronic supplementary material.

Supplementary file1 (PDF 8257 KB)

## Data Availability

All data supporting the findings of this study have been deposited in the Zenodo repository and are publicly available under an open-access license at 10.5281/zenodo.18068744. Zenodo ensures compliance with Ambio/Springer requirements by providing a persistent DOI for citation, long-term digital preservation, and interoperability with academic systems. The dataset includes raw and processed indicator data, analytical matrices, and accompanying metadata in standard formats to facilitate reuse and reproducibility.
