# Supplementary material for: Evaluating sustainability scenarios in coastal zones of Small Island Developing States (SIDS): A case study of the Dominican Republic
Source: Ambio. 2026 Feb 19;55(9):2233–49. doi: 10.1007/s13280-026-02345-7 (PMC13438532; doi:10.1007/s13280-026-02345-7)
Supplement: Supplementary file 1 [file 13280_2026_2345_MOESM1_ESM.pdf]

Supplementary Information

Appendix S – Figures

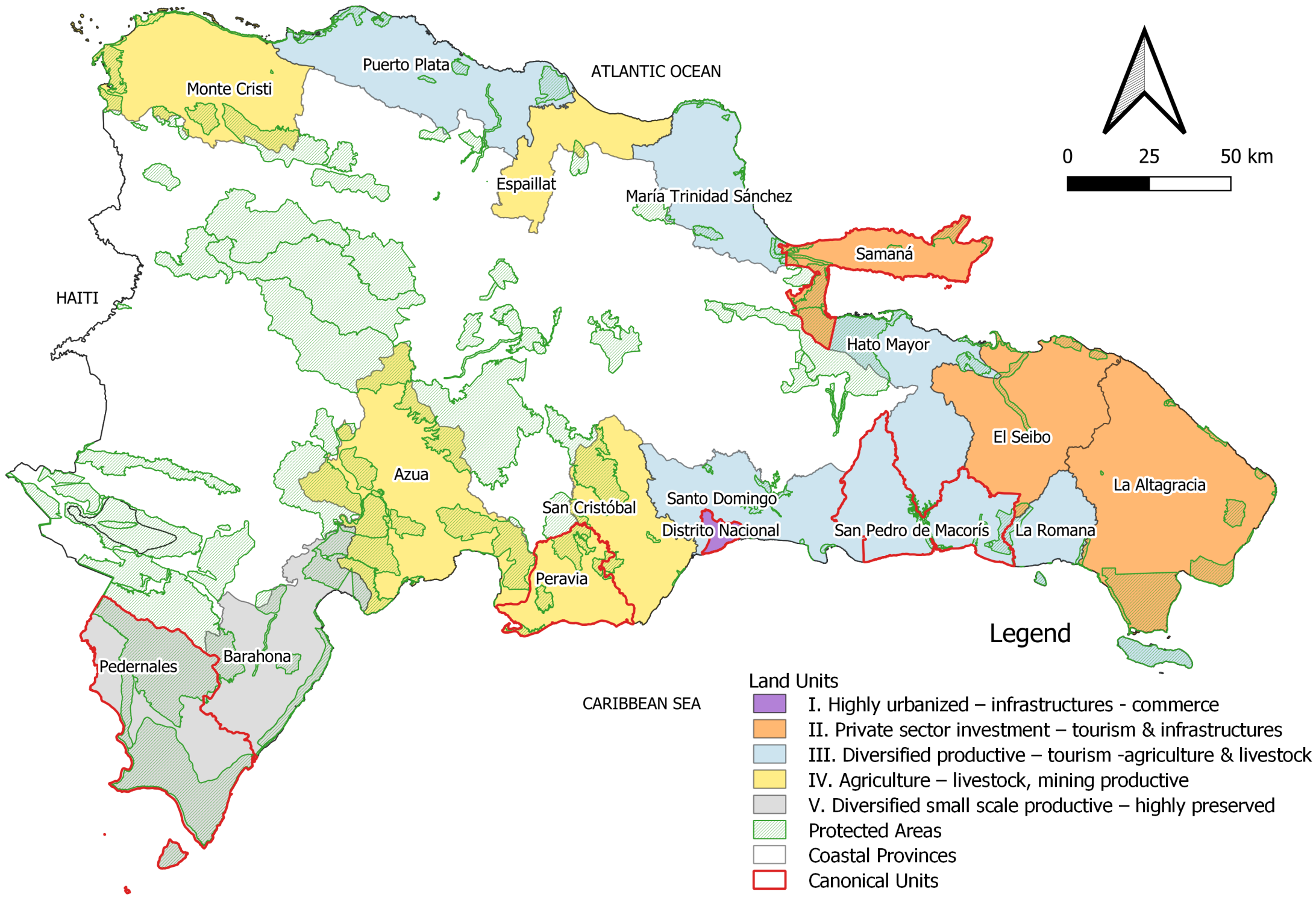

Figure S 1. Types of DEPCA and their canonical unit by group, distributed along the Dominican Republic coastline.

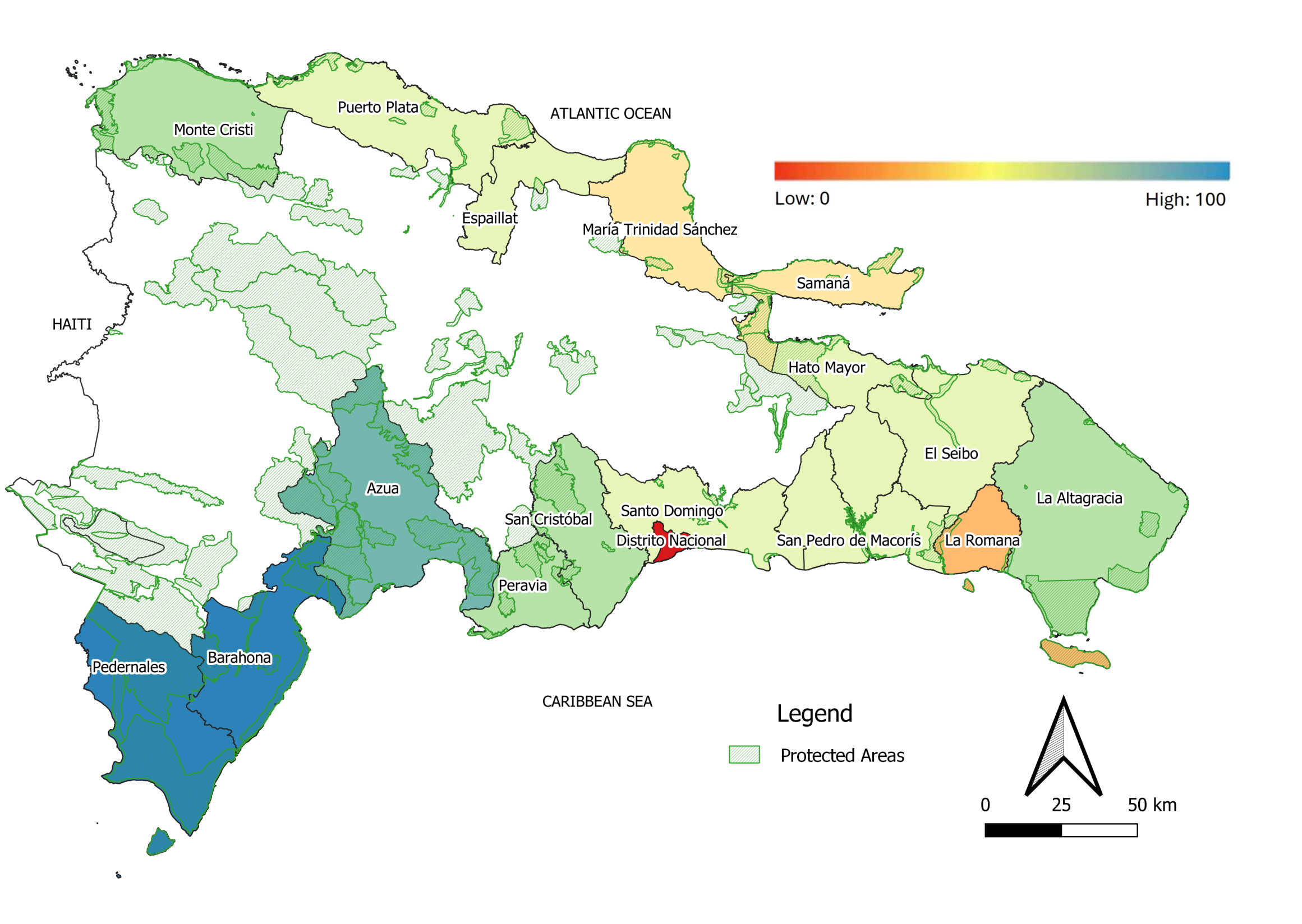

Figure S 2. Coastal provinces distribution according to the value obtained in the evaluation of the Ecological Dimension through a multivariate strategy. The scale has been made from 0 to 100 and for its representation it has been divided into eight colors, from red to blue.

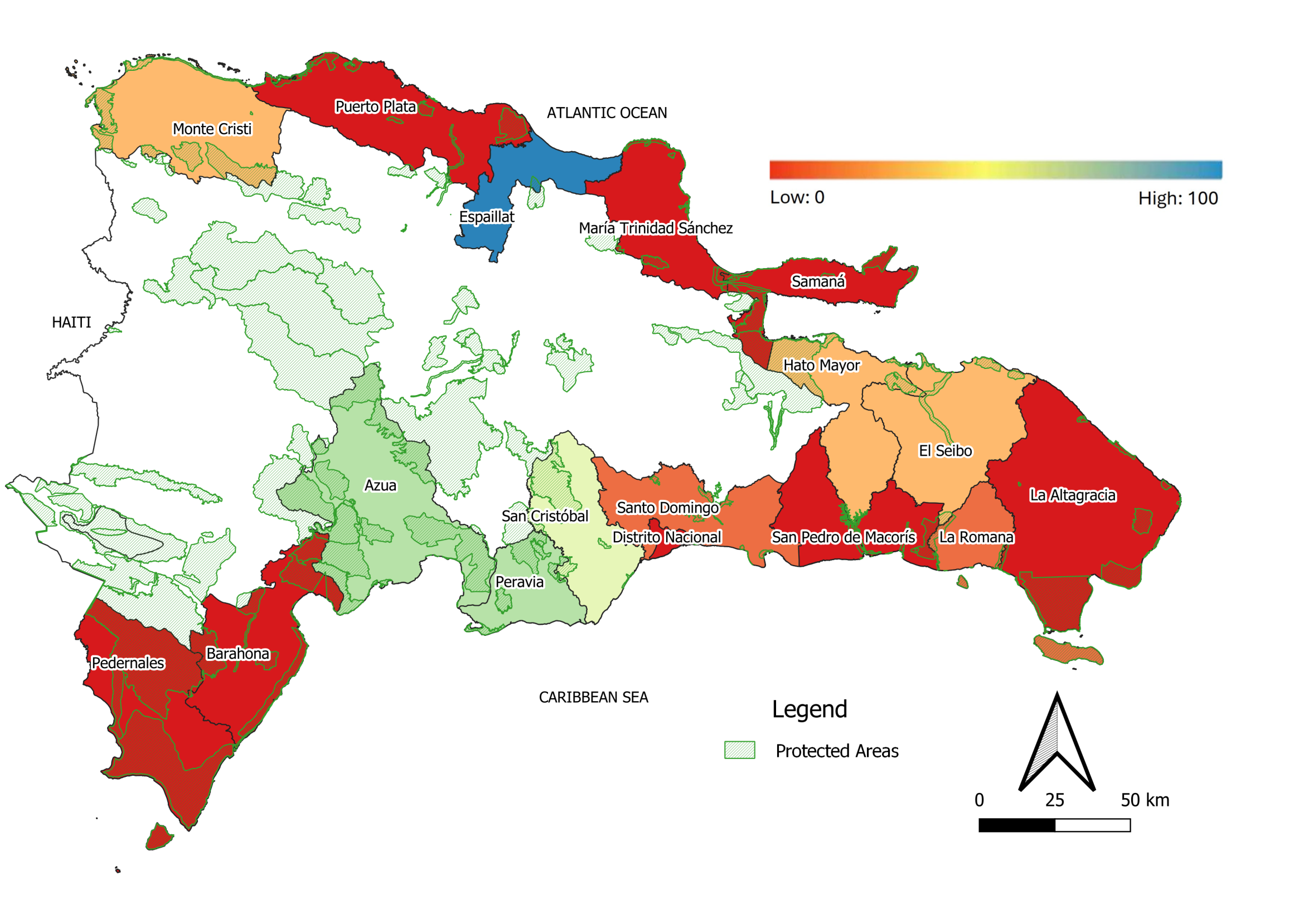

Figure S 3. Coastal provinces distribution according to the value obtained in the evaluation of the Productive Dimension through a multivariate strategy. The scale has been made from 0 to 100 and for its representation it has been divided into eight colors, from red to blue.

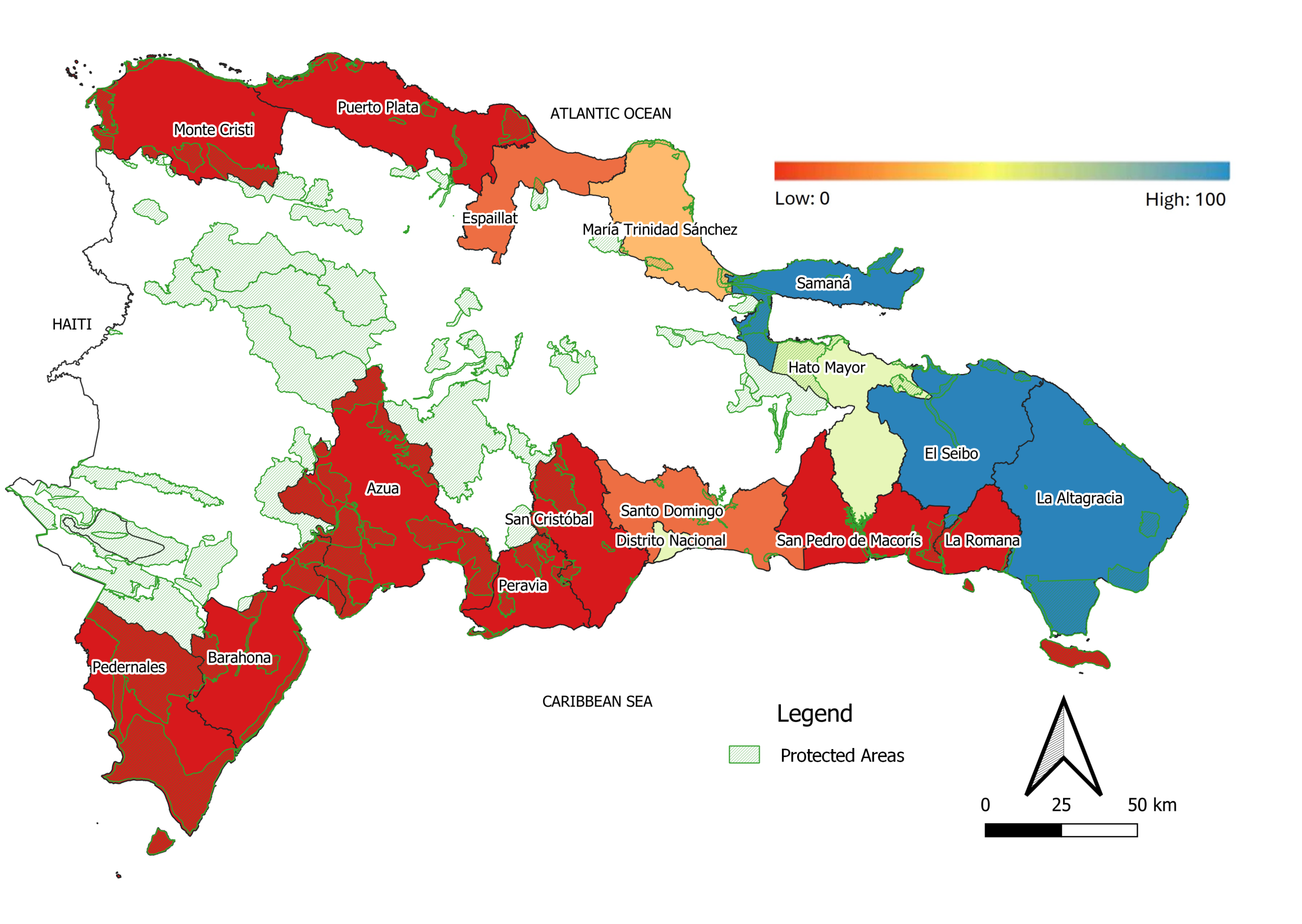

Figure S 4. Coastal provinces distribution according to the value obtained in the evaluation of the Economic Dimension through a multivariate strategy. The scale has been made from 0 to 100 and for its representation it has been divided into eight colors, from red to blue.

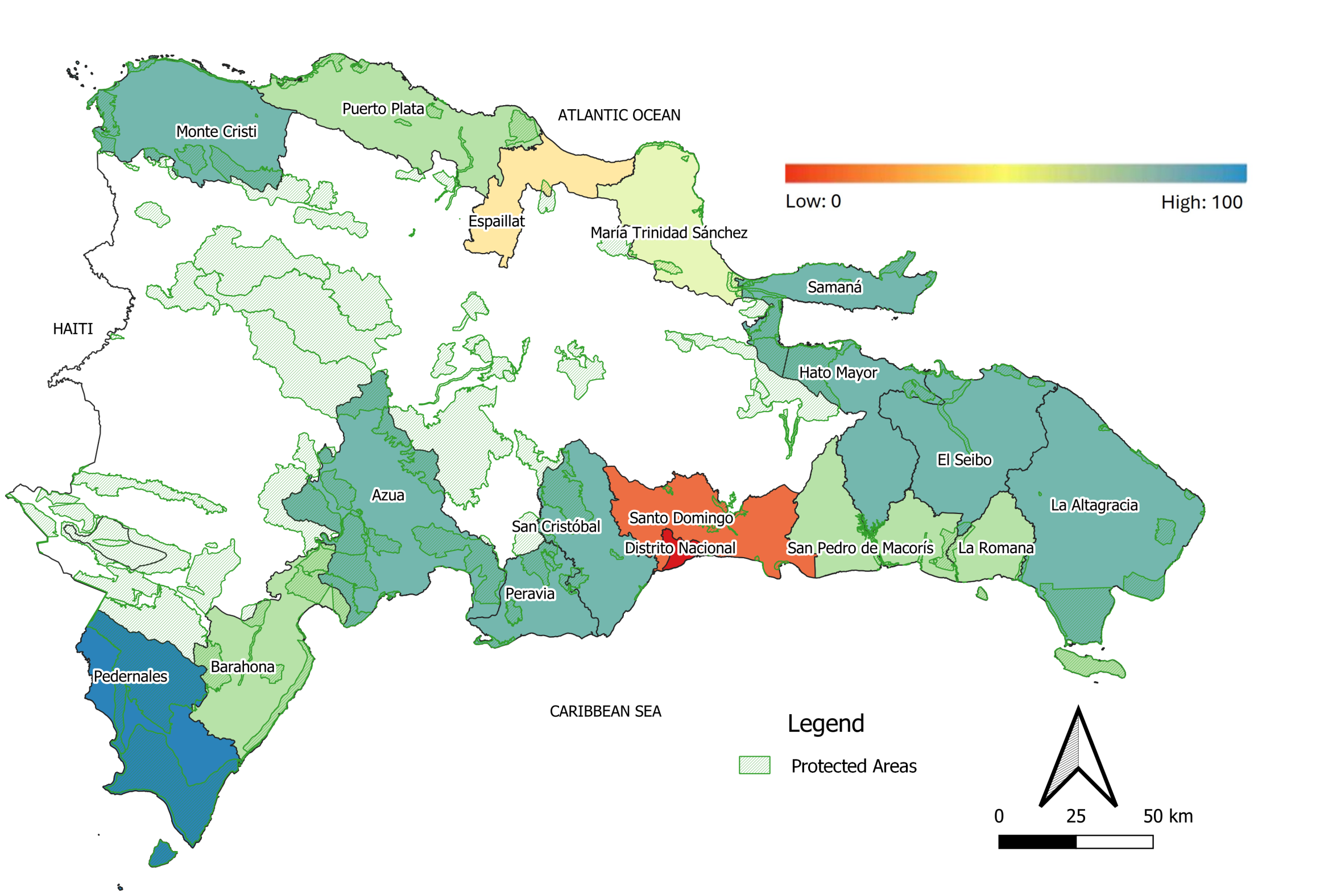

Figure S 5. Coastal provinces distribution according to the value obtained in the evaluation of the Cultural Dimension through a multivariate strategy. The scale has been made from 0 to 100 and for its representation it has been divided into eight colors, from red to blue.

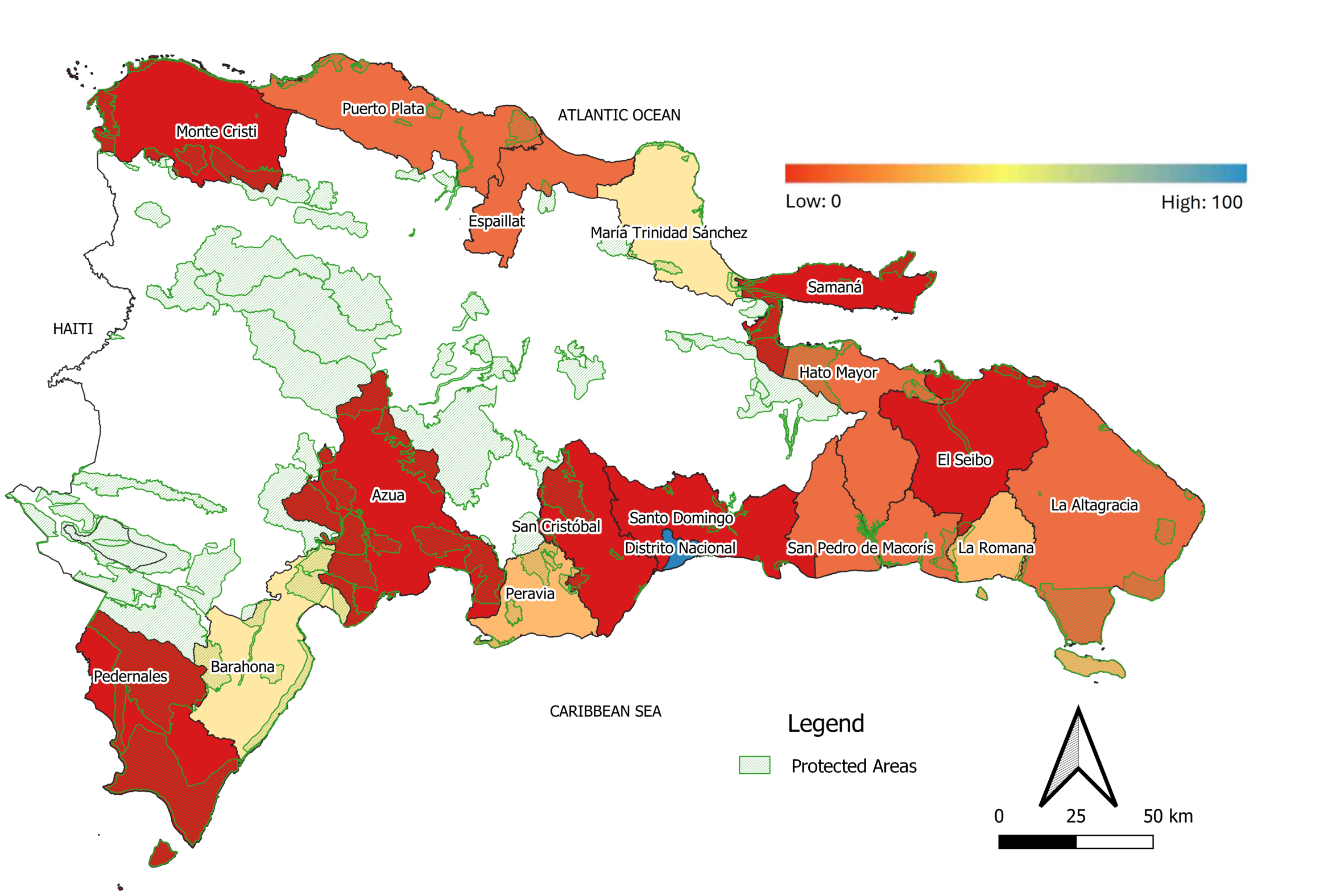

Figure S 6. Coastal provinces distribution according to the value obtained in the evaluation of the Social Dimension through a multivariate strategy. The scale has been made from 0 to 100 and for its representation it has been divided into eight colors, from red to blue.

Appendix S –Matrix and Tables

Appendix S. Matrix S 1. Variables per sustainability evaluative dimension **(**Ecological, *Economic, Productive, Cultural and Social)*

The ecological variables were obtained from the *Land Use and Cover Study* (Ministerio Ambiente, 2014). The geographic data, endemism, and invasive species from the *2012 Atlas of Biodiversity and Natural Resources of the Dominican Republic* (Ministerio Ambiente, 2012), and the *Sixth National Biodiversity Report* (Ministerio Ambiente, 2018a). The variables on the life zones were extracted from the study on the *Recognition and Evaluation of Natural Resources in the Dominican Republic* (OAS, 1967). The socioeconomic and productive data were obtained mainly from the 2010 *National Population and Housing Census* (ONE, 2022), *National Survey of Immigrants* (ONE, 2017), the *Provincial Profiles* (MEPyD, 2016) *and Provincial Development Plans* (MEPyD, 2017). While the cultural data was collected mainly from the document *Information on the Uses of Biological Resources and Traditional Knowledge, Innovations and Practices Associated with Genetic Resources* (Ministerio Ambiente, 2018b).

Evaluative dimensions	Carrying Capacity																																								
**Ecological** (ecosystems) (27 variables)	Balanced biophysical system. High coverage of Natural vegetation (slightly transformed):																												Healthy agrosystems. Complex agricultural landscapes. connectivity. Structure												
													Life zones (% of province)																												
Objective: To know the sustainability capacity (integrity, operation, structure) of the ecological systems of the provinces, in addition to their value for conservation	Area in km²	Forest cover (% total area of the province)	Types of forest in the province (No.)	Lakes and lagoons (%),	Beaches and dune systems (nº)	Length of the coast (km)	Rainfall (average mm/year)	Altitudinal range (masl)	Hydrographic basins (km²)	Average annual province temperature (degrees Celsius)	Average annual high temperature province (degrees Celsius)	Average annual low temperature province (degrees Celsius)	Subtropical Thorny Forest (%)	Subtropical Dry Forest (%)	Subtropical Moist Forest (%)	Subtropical Wet Forest (%)	Lower Montane Moist Forest (%)	Montane Wet Forest (%)	Lower Montane Wet Forest (%)	Subtropical Rain Forest (%)	Transitional Moist Forest to Very Moist Subtropical Forest (%)	Transitional Moist Forest to Lower Montane Dry Forest (%)	Transitional Moist Forest to Subtropical Dry Forest (%)	Transitional Moist Forest to Subtropical Rainforest (%)	Transition Very Moist Forest to Lower Montane Rainforest (%)	Transitional dry forest to Subtropical humid forest (%)	Types of life zones in the province (nº)	Soils productive capacity (% of Soil types I to VI of the surface of the province)	Water use pressure of the basins (%)	Hurricanes threat (Index)	River floods threat (Index)	Urban Flood Threat (Index)	Agricultural Drought Threat (Index)	Water Scarcity Threat (Index)	Surge - Coastal Flooding Threat (Index)	Landslide Threat (Index)	Forest Fire Threat (Index)	Seismic Threat (Index)	Total Threats (nº)	Physical hazards present in the province (nº)	Environmental Vulnerability Index
**Provinces**																																									
Distrito Nacional	91.58	16.3%	1	0.0%	5	13.89	1,500	71-0	48.00	26.3	30.7	21.9	0%	0.0%	15.3%	0	0.0%	0%	0.0%	0%	0%	0%	0%	0%	0.0%	0.0%	1	100.0%	32.5%	4	4	4	0	3	3	2	0	3	20	7	5
Santo Domingo	1,302.20	31.6%	2	0.0%	4	57.04	1,500	800-0	1302.2	26.1	30.5	21.7	0%	1.0%	92.3%	6.7%	0.0%	0%	0.0%	0%	0%	0%	0%	0.03%	0.0%	0.0%	4	94.9%	32.5%	4	4	4	1	3	3	3	4	3	26	9	5
Montecristi	1,885.81	32.4%	3	0.3%	10	137.56	592	700-0	1560.5	26.5	31.7	21.4	0%	94.4%	3.7%	0.0%	0.0%	0%	0.0%	0%	0%	0%	0.6%	0.00%	0.0%	1.2%	4	83.8%	96.1%	1	4	4	3	3	3	3	4	4	25	9	3
Puerto Plata	1,805.63	37.9%	3	0.02%	38	179.66	1,816	1200-0	1704.4	25.3	29.6	21.0	0%	3.7%	69.3%	25.5%	0.0%	0%	0.6%	0%	0.4%	0%	0.0%	0.52%	0.0%	0.0%	6	45.5%	14.3%	3	4	4	2	3	1	4	4	3	25	9	3
Espaillat	842.96	33.1%	2	0.01%	13	39.26	1,157	900-0	812.3	25.7	31.2	20.1	0%	0.5%	76.2%	22.9%	0.0%	0%	0.0%	0%	0.0%	0%	0.0%	0%	0.3%	0.0%	4	38.6%	14.3%	3	4	4	3	3	1	4	4	4	26	9	4
Maria Trinidad Sanchez	1,206.50	21.2%	2	0.1%	24	95.97	1,823	700-0	1198.0	26.0	30.3	21.7	0%	0.0%	52.3%	43.2%	0.0%	0%	0.0%	0%	4.5%	0%	0.0%	0%	0.0%	0.0%	3	72.9%	57.7%	3	4	4	1	3	2	3	4	4	24	9	3
Samana	862.82	37.6%	3	0.0%	40	223.43	2,000	500-0	846.2	26.0	31.1	21.0	0%	0.0%	65.2%	36.7%	0.0%	0%	0.0%	0%	0.0%	0%	0.0%	0%	0.0%	0.0%	2	26.2%	57.7%	3	4	3	1	3	3	3	4	4	24	9	3
Hato Mayor	1,319.30	28.1%	2	0.0%	6	84.25	1,642	500-0	1265.6	25.7	31.9	19.5	0%	43.7%	52.8%	0.0%	0.0%	0%	0.0%	0%	3.2%	0%	0.0%	0%	0.0%	0.0%	3	56.7%	19.6%	1	3	4	1	3	3	3	4	1	22	9	5
El Seibo	1,788.41	12.2%	2	0.6%	10	71.23	1,385	700-0	1326.3	25.7	31.7	19.7	0%	0.0%	66.2%	32.9%	0.0%	0%	0.0%	0.8%	0.0%	0%	0.0%	0%	0.0%	0.0%	3	58.5%	19.6%	1	1	4	4	3	3	3	4	1	23	9	5
La Altagracia	2,998.43	26.0%	2	0.1%	23	217.95	1,329	700-0	623.3	26.3	31.0	21.7	0%	45.4%	48.9%	0.0%	0.0%	0%	0.0%	0%	0.0%	0%	0.0%	0%	0.0%	3.5%	3	48.4%	19.6%	4	4	4	3	3	4	3	4	1	29	9	5
La Romana	652.12	19.4%	2	0.4%	5	24.92	1,539	100-0	537.5	26.0	31.3	20.8	0%	3.8%	85.9%	0.0%	0.0%	0%	0.0%	0%	0.0%	0%	10.3%	0%	0.0%	0.0%	3	83.8%	19.6%	4	2	2	1	3	4	2	4	1	22	9	6
San Pedro de Macoris	1,254.33	16.5%	2	0.2%	12	54.46	1,012	200-0	962.9	26.9	31.2	22.7	0%	2.8%	95.2%	0.0%	0.0%	0%	0.0%	0%	0.0%	0%	1.9%	0%	0.0%	0.0%	3	99.0%	32.5%	4	4	4	1	3	3	2	4	1	25	9	6
San Cristobal	1,240.63	44.8%	1	0.0%	6	32.36	1,500	1500-0	1145.3	26.0	31.4	20.7	0%	15.2%	45.5%	26.5%	0.0%	0.8%	11.4%	0%	0.0%	0%	0.0%	0.8%	0.0%	0.0%	6	31.5%	32.5%	4	4	4	2	3	3	4	4	3	28	9	6
Peravia	785.21	43.8%	4	0.0%	11	68.33	965	1700-0	743.9	27.6	32.8	22.4	5.6%	62.2%	18.0%	0.0%	10.5%	0%	1.5%	0%	0.6%	0%	0.0%	0%	0.0%	1.7%	7	47.7%	32.5%	4	4	4	3	3	3	4	4	3	29	9	5
Azua	2,682.54	48.8%	4	0.0%	8	86.3	696	2500-0	2366.9	27.4	32.2	21.4	13.3%	42.6%	12.8%	0.0%	25.2%	0.2%	3.5%	0%	0.4%	0.3%	0.0%	0%	0.2%	0.9%	10	34.6%	79.4%	4	4	4	2	3	3	4	4	3	28	9	4
Barahona	1,660.19	58.6%	4	1.2%	16	86.77	1,071	1600-0	664.2	26.6	30.5	22.6	2.6%	25.0%	46.4%	3.1%	8.1%	0.0%	14.4%	0%	1.1%	0%	0.0%	0%	0.0%	0.2%	8	41.5%	79.4%	4	4	4	4	3	3	4	4	3	30	9	4
Pedernales	2,080.50	72.2%	4	1.6%	19	130.59	402	2300-0	1889.1	27.6	33.7	21.5	2.4%	48.2%	9.7%	0.7%	5.5%	1.1%	9.8%	0%	0.0%	0%	3.8%	0%	0.5%	0.0%	9	37.1%	79.4%	4	1	2	4	3	3	3	4	1	24	9	4

Evaluative dimensions	Conservation value						
**Ecological (ecosystems) (27 variables)**	Maintenance of biodiversity, particularly threatened species					Protected area with national and international protection category (IUCN	
Objective: To know the sustainability capacity (integrity, operation, structure) of the ecological systems of the provinces, in addition to their value for conservation	Migratory species refuge (nº)	Turtle nesting site (nº)	Estuaries (nº)	Endemic species (nº)	Invasive species (nº of identified species)	Number of protected areas (Includes all categories: I to VI - IUCN)	Protected areas proportion (% total area of the province)
**Provinces**							
Distrito Nacional	1	3	0	2	4	3	15.3%
Santo Domingo	3	5	0	4	4	7	3.5%
Montecristi	7	3	1	13	3	8	16.9%
Puerto Plata	4	5	0	62	3	7	9.5%
Espaillat	1	0	1	3	2	1	1.4%
Maria Trinidad Sanchez	6	4	1	8	3	9	9.1%
Samana	5	6	1	166	3	5	38.6%
Hato Mayor	2	2	1	174	3	1	15.7%
El Seibo	4	1	1	96	3	4	3.2%
La Altagracia	4	10	0	85	5	9	15.3%
La Romana	2	2	1	84	8	6	22.4%
San Pedro de Macoris	1	3	2	84	3	5	6.2%
San Cristobal	3	2	0	18	6	4	44.7%
Peravia	4	5	0	11	3	6	21.0%
Azua	2	4	1	66	4	5	40.8%
Barahona	5	4	1	934	6	5	31.9%
Pedernales	4	10	0	1054	5	4	60.3%

**Evaluative dimensions**	**Economic specialization**											**Profitability**			
**Economic** (conventional economic system) (13 variables)	Products in high demand, easily marketable				Independence from external support							Well-structured, effective marketing network		Products with very high profitability, with high added value	
Objective: Securing local and national livelihoods; including tourism, trade, MSMEs, exports and mining.	Economically active population (%)	Tourist docks (nº of non-resident foreign arrivals)	Airports (nº of non-resident foreign arrivals)	% of the population employed in tourism and related services	Nº of free zone parks in operation	Nº of free zone companies in operation	Nº of employees in free zones	National exports, Free Zones and others (in thousands of USD)	National exports, Free Zones and others (Kilograms)	Cumulative investment of Free Zone companies (USD)	International maritime cargo, loaded and unloaded per year (mt)	Nº of formal MSME initiatives - in province (less than 10 employees)	Private sector investment in the construction sector (USD)	Nº of mining concessions	Volume of production of metallic and non-metallic minerals extracted (mt)
**Provinces**															
Distrito Nacional	25.1%	21,358	0	29.6%	5	44	12,788	427,730.50	168,257,678	149,769,780.50	0	14,193	$364,320,957.94	0	-
Santo Domingo	28.2%	0	973,737	29.6%	12	139	39,503	5,644,207 .3	1,451,981,147	1,697,078,839.50	11765800	4740	$428,427,604.50	21	633,083.10
Montecristi	26.7%	0	0	29.3%	1	3	472	117,470 .7	263,324,521	$ 9,883,578.00	458633	138	$1,894,062.43	5	21,344.00
Puerto Plata	22.6%	64,024	389,706	31.8%	3	17	1,321	458,633 .7	102,956,798	$ 17,978,398.50	1034787	1136	$3,393,312.63	10	64,331.00
Espaillat	21.8%	0	0	30.1%	1	16	2,793	0	0	$ 58,721,544.20	0	230	$13,341,515.66	1	3,455.00
María Trinidad Sánchez	33.7%	0	0	28.4%	1	2	72	0	0	$ 8,276,346.70	0	393	$2,299,821.13	2	8,750.00
Samaná	19.5%	0	81,868	32.0%	1	1	4	214.400	83,672	$ 810,192.70	0	313	$21,574,951.11	17	157,045.64
Hato Mayor	30.1%	0	0	26.8%	1	1	58	0	0	$ 300,000.00	0	136	$233,207.37	19	427,471.20
El Seibo	24.9%	0	0	21.0%	1	2	132	0	0	$ 811,296.30	0	89	$13,951,688.93	6	33,943.00
La Altagracia	34.1%	0	3,476,451	43.0%	1	6	2469	26,785.10	20,205.90	$ 27,368,354.40	0	670	$333,799,335.89	27	277,001.42
La Romana	26.7%	18,066	193,020	33.0%	2	15	8,852	80,912.00	227,087,663	$ 314,618,662.10	331328	872	$10,677,310.55	6	281,576.92
San Pedro de Macorís	23.3%	0	0	26.2%	3	44	11,859	66,429.10	591,754,479	$ 251,322,317.20	2046983	670	$29,204,126.47	22	122,666.40
San Cristóbal	22.1%	0	0	24.5%	9	66	24,008	28,201.00	183,234,774	$ 768,297,254.90	5740157	452	$10,005,930.79	35	1,294,621.89
Peravia	19.2%	0	0	30.1%	1	9	890	0	0	$ 76,417,280.70	1664903	253	$6,202,318.89	10	26,092.78
Azua	24.1%	0	0	22.1%	1	2	115	352.5	24,642	$ 2,010,139.00	206410	172	$190,944.16	15	170,000.00
Barahona	22.0%	0	0	20.5%	2	4	1,469	0	0	$ 49,920,843.00	277384	156	$335,733.16	32	232,417.78
Pedernales	28.8%	0	0	19.3%	1	1	677	0	0	$ 15,000,000.00	9475	13	$5,314.44	7	9,475.15

Evaluative dimensions	Ecological coherence													Productive efficiency													
**Productive** (production system, management, technology, planning) (45 variables)	Sound (prudent) extraction of natural resources							Products suitable for the attitude of the ecosystem						Diversity of species of wild flora and fauna that can be used sustainably	Efficiency and high productivity												
Objective: To ensure goods and services (agricultural and fishing products, trade, and tourist services; includes infrastructure, technology, and management systems)	Nº of fishermen	Small active artisanal fishing boats (nº),	Nº of landing ports - artisanal fishermen	Hotel rooms density (beds / km²)	Visit to protected areas (number of visitors/year)	Water demand in tourism sector (m3/year)	Cultivated area under protected/controlled environment (m²)	Nº of agricultural producers	Nº of livestock producers	Agricultural and subsistence crops (% surface area of the province)	Pastures for livestock (% surface area of the province)	Sugarcane plantations (% surface area of the province)	Net area of cultivation with irrigation (ha)	Nº of species of wild flora and fauna used for craftmanship	Houses (nº)	Gross Generated Energy (GWh)	Renewable Energy (Annual megawatt production)	Internet Access (% population with access to internet accounts in the province)	Free zone parks in operation (nº)	Use of public transport by the population (%)	Paved roads in urban areas of the province (%)	Commercial seaports (nº)	Tourist ports (nº)	Airports (nº)	Water for agricultural production (length of irrigation channels in km)	Nº of hotels	Tourist offer (nº of beds)
**Provinces**																											
Distrito Nacional	112	21	4	64.40	0	4,690,000	500	0	0	0.0%	0.0%	0.0%	0.0%	4	331,108	1,487.9	0	33.3%	5	44.8%	78.9%	1	1	0	0	113	5,902
Santo Domingo	331	50	10	1.61	128,035	1,916,000	226,400	7,282	2,744	24.1%	11.3%	11.7%	0.0%	9	756,183	5,732.8	97.2	17.2%	12	58.6%	61.1%	4	1	2	0	56	2,098
Montecristi	838	111	12	0.08	33,409	143,000	55,500	3,793	4,919	40.4%	7.1%	0.0%	25.4%	4	40,647	0	294.4	4.4%	1	20.6%	84.3%	1	0	0	79.8	9	155
Puerto Plata	2,653	107	19	7.48	212,544	13,803,000	77,425	8,420	3,856	25.8%	31.0%	3.1%	0.4%	6	116,606	876 .1	48	12.4%	3	21.1%	67.8%	3	2	1	10.44	162	13,852
Espaillat	714	4	9	0.18	0	48,000	905,671	8,812	3,070	39.3%	25.8%	0.0%	0.0%	2	70,561	0	0	8.9%	1	19.5%	67.6%	0	0	0	0	9	152
María Trinidad Sánchez	1,382	73	13	1.02	91,634	1,964,000	22,628	6,202	2,424	44.4%	35.1%	0.0%	21.7%	5	46,763	9.5	0.9	6.9%	1	39.7%	50.9%	0	0	0	126.6	39	1,292
Samaná	3,408	516	57	3.84	71,491	2,669,000	25,200	4,752	628	53.9%	6.9%	0.0%	0.0%	5	34,678	39.4	0	5.4%	1	24.60%	38.6%	1	1	3	0	71	3,278
Hato Mayor	685	144	6	0.05	45,256	33,000	8,312	6,106	439	34.5%	14.0%	34.5%	0.5%	6	28,482	0	0	5.5%	1	14.6%	76.4%	0	0	0	22.2	5	61
El Seibo	1,054	98	10	0.04	1,536	65,000	18,282	4,970	1,087	26.0%	43.4%	15.3%	0.0%	5	29,315	0	0	3.5%	1	17.80%	62.7%	0	0	0	0	4	65
La Altagracia	695	63	8	15.30	728,943	31,861,000	15,292	3,910	1,690	12.9%	46.9%	9.2%	0.6%	9	109,096	317.5	0	10.0%	1	30.1%	77.1%	1	0	1	11.8	123	46,046
La Romana	200	7	4	2.53	841,933	727,000	63,200	1,221	213	3.6%	25.7%	30.8%	0.0%	4	79,220	174.6	0	11.4%	2	69.9%	71.9%	1	1	1	0	32	1,653
San Pedro de Macorís	525	59	8	2.01	20,092	3,510,000	7,300	3,226	636	15.2%	8.1%	49.8%	0.0%	8	98,174	2,259.30	223.8	10.2%	3	37.2%	64.4%	2	0	0	0	35	2,529
San Cristóbal	247	47	4	0.06	11,327	39,000	25,000	16,093	3,955	35.4%	12.3%	2.1%	3.0%	6	168,014	385.1	112.2	9.0%	9	24.2%	78.9%	1	0	0	51.9	5	79
Peravia	419	76	10	0.14	5,535	73,000	157,752	4,855	1,717	29.4%	4.3%	3.5%	9.1%	6	57,592	462	303 .97	7.6%	1	27.6%	73.2%	2	0	0	129.7	5	114
Azua	499	83	7	0.01	0	26,000	67,984	14,855	1,719	31.9%	1.6%	0.0%	6.7%	6	61219	488 .7	124 .3	4.1%	1	29.4%	90.3%	1	0	0	241.2	2	26
Barahona	680	74	15	0.29	0	487,000	21,142	8,046	1,164	18.9%	6.9%	5.6%	2.8%	15	54,378	383.7	570.7	5.6%	2	26.1%	58.8%	1	0	1	149.7	21	510
Pedernales	487	40	9	0.03	24,417	58,000	285	1,688	367	11.1%	2.9%	1.7%	0.2%	5	7,787	0	209	3.1%	1	14.9%	43.3%	1	0	0	21.3	5	58

Evaluative dimensions	**Heritage**				**Knowledge**							
**Cultural** (Ancestral knowledge and resilience to challenges) (15 variables)	Important natural and cultural heritage		Technological capital. Adequate technology, modernized knowledge and with scientific bases		Adapted knowledge about resources, empirical knowledge, rural culture			Training and learning; ability to face new challenges				
Objective: To ensure the preservation and appreciation of the ancestral heritage and traditional knowledge of local communities	Sites and cultural manifestations under protection -NSPA - RAMSAR - UNESCO - ICOMOS (nº)	Caves with Archaeological - Cultural importance (nº)	Museums and cultural centers (nº)	Public libraries (nº)	Valued folkloric manifestations (nº)	Folkloric dances (rituals and occasions) preserved (nº)	Crafts in ancestral materials and techniques preserved (nº)	Nº of enrolled in high school	Enrolled in technical-higher education (nº)	Nº of people with a university education	Women with a university education (%)	Positions in local governments held by women (%)
**Provinces**												
Distrito Nacional	4	51	40	75	6	4	9	77,488	1,935	257,590	56%	37.2%
Santo Domingo	10	41	11	13	12	6	7	354,276	7,262	634,643	58%	35.3%
Montecristi	9	5	3	9	3	3	2	10,878	0	6,866	63%	43.9%
Puerto Plata	9	13	11	7	2	3	5	39,949	119	28,928	59%	32.3%
Espaillat	1	3	1	8	2	1	5	24,934	4	30,152	60%	31.6%
María Trinidad Sánchez	9	10	0	8	2	1	2	14,777	0	13,736	63%	42.4%
Samaná	6	31	1	4	3	4	7	12,623	1	7,356	63%	35.7%
Hato Mayor	1	24	1	7	2	3	3	12,804	0	7,462	64%	40.0%
El Seibo	4	6	1	5	2	2	4	8,750	0	4,583	62%	37.2%
La Altagracia	11	53	8	7	2	3	5	39,024	181	22,660	56%	36.7%
La Romana	6	11	3	5	2	2	6	34,966	0	24,009	59%	41.9%
San Pedro de Macorís	6	14	4	14	2	2	4	40,361	0	29,140	60%	33.8%
San Cristóbal	7	8	5	7	13	3	6	89,883	0	56,111	47%	33.9%
Peravia	6	0	2	6	11	4	2	20,940	0	11,095	67%	40.3%
Azua	6	6	2	4	12	5	5	23,017	0	14,283	64%	33.0%
Barahona	5	4	1	7	6	4	9	24,776	97	19,752	60%	28.6%
Pedernales	5	36	0	2	1	1	1	3,132	0	1,215	59%	37.5%

**Social** (social and human well-being) (48 variables)	Social integrity, solidarity, identity, and sense of belonging											Level of equity in the distribution of wealth					Ability to provide socially appreciated services								
Objective: To ensure the livelihoods and well-being of the population, meeting basic needs.	Province inhabitants (nº)	Average household size (nº of people)	Access to drinking water inside the home (% population)	Access to garbage collection by municipality (%)	Households without sanitary service (%))	Households with paved access roads (%)	Access to electricity (%)	Use of charcoal or firewood as fuel for cooking (% households)	Registered mobile phone users (% population)	Households with access to fixed telephony (%)	Households exposed to natural disasters risk (%)	Province Human Development Index (HDI)	Households in general poverty (%)	Households in extreme multidimensional poverty (%)	Unemployment (%)	Illiteracy (%)	Schools (public and private) (nº)	Total (Public and private) universities (nº)	Private universities (%)	Universities (nº of students enrolled)	Students enrolled in public universities (%)	Enrolled in technical-superior careers (nº)	Public primary care health centers (nº)	Public hospitals (nº)	Physicians/ 10,000 inhab. (ag)	Public landfills (nº)
**Provinces**																										
Distrito Nacional	1,484,789	3.3	63.5%	86.4%	1.4%	75.0%	99.7%	0.90%	92.2%	48.6%	25.6%	0.734	26.70%	4.70%	6.20%	2.6%	914	26	96.2%	261,685	44%	1,935	128	27	37.1	0
Santo Domingo	2,995,211	3.6	51.5%	78.3%	3.5%	56.1%	99.2%	3.10%	86.1%	33.0%	21.1%	0.487	33.60%	6.90%	16.30%	3.0%	2,716	6	83.3%	38,168	17%	7,262	142	28	10.6	8
Montecristi	135,710	3.2	41.3%	50.9%	10.0%	27.8%	84.0%	23.70%	83.2%	11.0%	24.8%	0.387	61.30%	23.50%	4.90%	10.3%	157	1	100.0%	438	0%	0	36	5	13.5	8
Puerto Plata	490,733	3.3	47.1%	72.1%	5.7%	45.8%	94.5%	16.20%	89.5%	16.7%	33.6%	0.558	37.20%	9.50%	5.60%	7.1%	488	3	66.7%	9,584	35%	119	36	13	8.2	6
Espaillat	390,478	3.6	49 .5%	65.5%	5.2%	35.4%	96.8%	11.60%	79.6%	16.0%	31.2%	0.409	34.00%	6.20%	19.50%	6.3%	269	3	100.0%	6,439	0%	4	41	11	9.3	4
María Trinidad Sánchez	140,784	3.5	36.4%	56.6%	9.2%	40.4%	95.6%	14.30%	86.7%	10.6%	17.3%	0.471	42.00%	7.90%	12.00%	6.8%	263	2	50.0%	11,529	52%	0	29	5	10.5	5
Samaná	168,265	3.5	31.8%	53.7%	11.1%	42.6%	76.3%	15.30%	78.2%	6.20%	22.3%	0.487	48.90%	13.70%	11.80%	8.7%	144	1	0.0%	280	100%	1	19	4	12.3	5
Hato Mayor	89,578	3.5	22.5%	64.6%	13.6%	35.5%	89.2%	24.30%	89.3%	10.0%	15.5%	0.417	62.40%	21.70%	8.60%	8.2%	136	1	100.0%	3,927	100%	0	24	3	18.5	1
El Seibo	115,889	3.4	27.4%	48.9%	22.5%	37.1%	72.4%	37.30%	86.2%	8.10%	25.0%	0.316	70.70%	32.80%	18.20%	8.1%	171	0	0.0%	0	0	0	23	2	13.7	7
La Altagracia	335,677	3.1	8.7%	67.7%	5.3%	38.0%	91.0%	9.60%	93.9%	10.50%	13.6%	0.488	51.80%	17.10%	13.80%	3.6%	273	6	83.3%	11,795	70%	181	40	3	7.4	4
La Romana	330,587	3.5	41.0%	76.7%	5.5%	60.6%	94.5%	6.70%	92.2%	17.90%	11.2%	0.419	45.30%	11.90%	13.90%	4.2%	296	3	66.7%	15,559	5%	0	19	2	8.3	1
San Pedro de Macorís	418,850	3.5	30.8%	75.8%	10.2%	42.4%	96.7%	11.50%	93.3%	17.60%	13.4%	0.522	47.50%	11.30%	15.40%	3.4%	306	3	66.7%	10,606	28%	0	37	4	17.7	1
San Cristóbal	859,741	3.8	37.4%	67.9%	6.0%	45.9%	97.9%	13.50%	77.1%	16.80%	34.4%	0.419	36.20%	6.40%	19.60%	4.6%	683	4	75.0%	5,665	71%	0	83	8	8.4	6
Peravia	298,747	3.7	36.7%	73.6%	6.1%	58.8%	97.8%	15%	76.2%	21.50%	26.2%	0.413	47.30%	10.90%	23.20%	7.5%	152	2	50.0%	12,572	15%	0	36	3	10.0	7
Azua	256,981	3.9	27.1%	80.1%	15.9%	40.0%	91.9%	26.80%	86.3%	9.40%	14.7%	0.301	65.20%	24.40%	17.30%	16.1%	249	1	100.0%	2,530	0%	0	48	4	11.4	19
Barahona	226,898	3.9	29.1%	70.2%	14.3%	62.6%	91.1%	28.0%	85.7%	11.60%	22.1%	0.271	65.10%	26.80%	22.90%	6.6%	224	2	50.0%	17,335	73%	97	48	6	11.9	15
Pedernales	38,941	3.4	22.9%	52.4%	24.8%	52.5%	67.6%	42.30%	82.2%	3.50%	20.8%	0.145	74.60%	44.60%	6.80%	13.0%	26	1	100.0%	235	0%	0	6	1	14.1	3

**Social** (social and human well-being) (48 variables)	Maintaining a stable and balanced demographic																	
Objective: To ensure the livelihoods and well-being of the population, meeting basic needs.	Population density per km²	Houses (nº)	Urban population (%)	Nº of Municipalities	Nº of Municipal Districts	Nº of Neighborhoods	Populations with less than 10,000 inhabitants (nº)	Populations from 10,000 to 50,000 inhabitants (nº)	Populations of 50,000 to 100,000 inhabitants (nº)	Populations of more than 100,000 inhabitants (nº)	Population growth (% average annual growth rate)	Immigration rate (%)	Health index	Registered births of mothers under 20 years of age (%)	Adolescent pregnancy rate (%)	Infant mortality rate (Rate per 100,000 live births)	Population average age (under 34 years)	Percentage of aging population (over 65 years)
**Provinces**																		
Distrito Nacional	10,538	331,108	100%	1	0	70	0	0	0	1	0.69%	10.9%	0.516	17.40%	14.9%	13.7	64.0%	7.2%
Santo Domingo	1,823	756,183	88%	7	8	93	2	4	5	4	3.40%	7.2%	0.417	17.10%	18.1%	18.4	64.0%	4.5%
Montecristi	58	40,647	53%	6	4	54	6	4	0	0	-0.16%	18.9%	0.381	29.00%	25.9%	22 .9	47.0%	7.9%
Puerto Plata	178	116,606	58%	9	12	91	14	6	0	1	0.36%	7.8%	0.477	24.50%	19.3%	17 .1	47.0%	7.0%
Espaillat	275	70,561	45%	4	11	115	8	6	1	0	0.38%	6.0%	0.334	25.80%	18.4%	16 .4	61.9%	7.6%
María Trinidad Sánchez	117	46,763	53%	4	6	97	2	8	0	0	0.48%	3.4%	0.467	26.30%	22.3%	14.0%	63.2%	7.3%
Samaná	118	34,678	44%	3	3	43	2	4	0	0	1.31%	7.6%	0.434	28.90%	22.2%	8.4%	65.5%	6.3%
Hato Mayor	64	28,482	26%	3	4	50	5	2	0	0	-0.37%	5.8%	0.542	23.40%	20.6%	10.0	50.0%	7.8%
El Seibo	49	29,315	51%	2	5	47	4	3	0	0	-0.22%	15.3%	0.390	28.20%	23.4%	3.9	64.0%	8.5%
La Altagracia	91	109,096	78%	2	5	83	3	3	0	1	6.26%	15.8%	0.478	22.70%	26.2%	10.3	64.0%	4.3%
La Romana	376	79,220	94%	3	2	76	1	2	1	1	1.46%	13.1%	0.280	20.00%	17.5%	12.2	67.0%	5.2%
San Pedro de Macorís	232	98,174	84%	6	2	139	3	4	0	1	-0.47%	5.7%	0.528	20.90%	17.7%	11.7	66.0%	5.7%
San Cristóbal	459	168,014	52%	8	6	94	3	7	3	1	0.87%	2.7%	0.394	23.90%	20.4%	11.1	68.0%	5.1%
Peravia	235	57,592	68%	2	11	81	9	3	1	0	1.07%	3.9%	0.386	25.90%	19.9%	10.4	65.3%	6.5%
Azua	80	61219	77%	10	22	105	28	2	1	0	0.33%	4.1%	0.314	26.10%	26.6%	11.1	67.0%	6.3%
Barahona	113	54,378	83%	11	11	148	16	5	1	0	0.55%	4.3%	0.199	26.60%	24.9%	11.8	68.0%	6.4%
Pedernales	15	7,787	64%	2	2	35	3	1	0	0	6.1%	10.8%	0.257	33.10%	27.1%	9.8	70.0%	4.9%

Table S 1.

Evaluative dimensions involved in the multi-criteria analysis of sustainability. The characteristics of each dimension and the variables selected for an estimation of their value in each DR coastal province, by means of multivariate (DCA) analysis, are indicated.

Evaluative dimensions[1]	***Characteristics/Objectives of the dimension***	***Variables***
Ecological (ecosystems) *(27 variables)*	To know the sustainability capacity (integrity, operation, structure) of the ecological systems of the provinces, in addition to their conservation value.	**Carrying Capacity:**<br><br>• *Balanced biophysical system. High coverage of Natural vegetation (slightly transformed):* Area in km², Forest cover (% total area of the province), Types of forest in the province (nº), Lakes and lagoons (%), Beaches and dune systems (nº), Length of the coast (km), Rainfall (average mm/year), Altitudinal range (MASL), Hydrographic basins (km²), Average annual province temperature (degrees Celsius), Average annual high temperature province (degrees Celsius), Average annual low temperature province (degrees Celsius) , Life zones - % of province (Subtropical Thorny Forest, Subtropical Dry Forest, Subtropical Moist Forest, Subtropical Wet Forest, Lower Montane Moist Forest, Montane Wet Forest, Lower Montane Wet Forest, Subtropical Rainforest, Transitional Moist Forest to Very Moist Subtropical Forest, Transitional Moist Forest to Lower Montane Dry Forest, Transitional Moist Forest to Subtropical Dry Forest, Transitional Moist Forest to Subtropical Rainforest, Transition Very Moist Forest to Lower Montane Rainforest, Transitional dry forest to Subtropical humid forest), Types of life zones in the province (nº)<br><br>• *Healthy agrosystems. Complex agricultural landscapes. connectivity. Structure:* Soils productive capacity (% of Soil types I to VI of the surface of the province), Water use pressure of the basins (%), Hurricanes threat (Index), River floods threat (Index), Urban Flood Threat (Index), Agricultural Drought Threat (Index), Water Scarcity Threat (Index), Surge - Coastal Flooding Threat (Index), Landslide Threat (Index), Forest Fire Threat (Index), Seismic Threat (Index), Total Threats (nº), Physical hazards present in the province (nº), Environmental Vulnerability Index.<br><br>**Conservation value:**<br><br>• *Maintenance of biodiversity, particularly threatened species:*<br>Migratory species refuge (nº), Turtle nesting site (nº), Estuaries (nº), Endemic species (nº), Invasive species (nº of identified species).<br>• *Protected area with national and international protection category (IUCN):*<br>Number of protected areas (Includes all categories: I to VI - IUCN), Protected areas proportion (% total area of the province)

[1] Adapted from Gómez Sal (2004)

Economic (conventional economic system) *(13 variables)*	Securing local and national livelihoods; including tourism, trade, MSMEs, exports and mining.	**<u>Economic specialization</u>**<br>• *Products in high demand, easily marketable*<br>Economically active population (%), Tourist docks (number of non-resident foreign arrivals), Airports (nº of non-resident foreign arrivals), % of the population employed in tourism and related services.<br>• *Independence from external support*<br>Nº of free zone parks in operation, Nº of free zone companies in operation, Nº of employees in free zones, National exports, Free Zones and others (in thousands of USD), National exports, Free Zones and others (Kilograms), Cumulative investment of Free Zone companies (USD), International maritime cargo, loaded and unloaded per year (mt).<br>**<u>Profitability</u>**<br>• *Well-structured, effective marketing network*<br>Nº of formal MSME initiatives – in the province (less than 10 employees), Private sector investment in the construction sector (USD)<br>•*Products with very high profitability, with high added value*<br>Nº of mining concessions, Volume of production of metallic and non-metallic minerals extracted (mt).
Productive (production system, management, technology, planning) *(45 variables)*	The way goods and services (agricultural and fisheries products, trade and tourism services; include infrastructure, technology and management systems) are obtained and secured.	**<u>Ecological coherence</u>**<br>• *Sound (prudent) extraction of natural resources*<br>Nº of fishermen, Small active artisanal fishing boats (nº), Nº of landing ports - artisanal fishermen, Hotel rooms density (beds/ km²), Visit to protected areas (number of visitors/year), Water demand in tourism sector (m3/year), Cultivated area under protected/controlled environment (m²).<br>• *Products suitable for the attitude of the ecosystem*<br>Nº of agricultural producers, Nº of livestock producers, Agricultural and subsistence crops (% surface area of the province), Pastures for livestock (% surface area of the province), Sugarcane plantations (% surface area of the province), Net area of cultivation with irrigation(ha).<br>**<u>Productive efficiency</u>**<br>• *Diversity of species of wild flora and fauna that can be used sustainably*<br>Nº of species of wild flora and fauna used for craftmanship<br>• *Efficiency and high productivity*<br>Houses (nº), Gross Generated Energy (GWh), Renewable Energy (Annual megawatt production), Internet Access (% population with access to internet accounts in the province), Free zone parks in operation (nº), Use of public transport by the population (%), Paved roads in urban areas of the province (%), Commercial seaports (nº), Tourist ports (nº), Airports (nº), Water for agricultural production (length of irrigation channels in km), Nº of hotels, Tourist offer (nº of beds)
Cultural (Ancestral knowledge and resilience to challenges) *(15 variables)*	To ensure the preservation and appreciation of the ancestral heritage and the useful and traditional knowledge of local communities	**<u>Heritage</u>**<br>• *Important natural and cultural heritage*<br>Sites and cultural manifestations under protection -NSPA - RAMSAR - UNESCO - ICOMOS (nº), Caves with Archaeological - Cultural importance (nº).

		• *Technological capital. Adequate technology, modernized knowledge and scientific bases.*<br><br>Museums and cultural centers (nº), Public libraries (nº)<br>**Knowledge**<br>• *Adapted knowledge about resources, empirical knowledge, rural culture*<br>Valued folkloric manifestations (nº), Folkloric dances (rituals and occasions) preserved (nº), Crafts in ancestral materials and techniques preserved (nº)<br>• *Training and learning; ability to face new challenges*<br>Nº of enrolled in high school, Enrolled in technical-higher education (nº), Nº of people with a university education, Women with a university education (%), Positions in local governments held by women (%).
Social (social and human well-being)<br>*(48 variables)*	Ensure the livelihoods and well-being of the population, meeting basic needs.	**Well-being, integration**<br>• *Social integrity, solidarity, identity and sense of belonging*<br>Province inhabitants (nº), Average household size (nº of people), Access to drinking water inside the home (% population), Access to garbage collection by municipality (%), Households without sanitary service (%), Households with paved access roads (%), Access to electricity (%), Use of charcoal or firewood as fuel for cooking (% households), Households with access to fixed telephony (%), Registered mobile phone users (% population), Households exposed to natural disasters risk (%)<br>• *Level of equity in the distribution of wealth*<br>Province Human Development Index (HDI), Households in general poverty (%), Households in extreme multidimensional poverty (%), Unemployment (%), Illiteracy (%)<br>**Strategic use of territory and resources**<br>• *Ability to provide socially appreciated services*<br>Schools (public and private) (nº), Total (Public and private) universities (nº), Private universities (%), Universities (nº of students enrolled), Students enrolled in public universities (%), Enrolled in technical-superior careers (nº), Public primary care health centers (nº), Public hospitals (nº), Physicians/ 10,000 inhab. (ag), Public landfills (nº).<br><br>• *Maintaining a stable and balanced demographic*<br>Population density per km^2, Houses (nº), Urban population (%), Nº of Municipalities, Nº of Municipal Districts, Nº of Neighborhoods, Populations with less than 10,000 inhabitants (nº), Populations from 10,000 to 50,000 inhabitants (nº), Populations of 50,000 to 100,000 inhabitants (nº), Populations of more than 100,000 inhabitants (nº), Population growth (% average annual growth rate), Immigration rate (%), Health index, Registered births of mothers under 20 years of age (%), Adolescent pregnancy rate (%), Infant mortality rate (Rate per 100,000 live births), Population average age (under 34 years), Percentage of aging population (over 65 years)

Table S 2.

Detrended Correspondence Analysis (DCA) Results. 1st Axis – (DCA1)

	Ecological Dimension	DCA1		Economic Dimension	DCA1		Productive Dimension	DCA1
**1**	Distrito Nacional	0.000	1	Distrito Nacional	0.970	1	Distrito Nacional	0.065
**2**	Santo Domingo	0.894	2	Santo Domingo	0.240	2	Santo Domingo	0.488
**3**	Montecristi	1.148	3	Montecristi	0.000	3	Montecristi	0.734
**4**	Puerto Plata	0.970	4	Puerto Plata	0.052	4	Puerto Plata	0.016
**5**	Espaillat	0.936	5	Espaillat	0.387	5	Espaillat	2.150
**6**	María Trinidad Sánchez	0.788	6	María Trinidad Sánchez	0.443	6	María Trinidad Sánchez	0.063
**7**	Samaná	0.719	7	Samaná	1.723	7	Samaná	0.037
**8**	Hato Mayor	0.883	8	Hato Mayor	0.864	8	Hato Mayor	0.694
**9**	El Seibo	0.979	9	El Seibo	1.700	9	El Seibo	0.733
**10**	La Altagracia	1.017	10	La Altagracia	1.670	10	La Altagracia	0.000
**11**	La Romana	0.538	11	La Romana	0.067	11	La Romana	0.355
**12**	San Pedro de Macorís	0.891	12	San Pedro de Macorís	0.068	12	San Pedro de Macorís	0.027
**13**	San Cristóbal	1.022	13	San Cristóbal	0.068	13	San Cristóbal	1.130
**14**	Peravia	1.166	14	Peravia	0.186	14	Peravia	1.500
**15**	Azua	1.340	15	Azua	0.215	15	Azua	1.511
**16**	Barahona	1.477	16	Barahona	0.080	16	Barahona	0.217
**17**	Pedernales	1.642	17	Pedernales	0.067	17	Pedernales	0.260

	**Cultural Dimension**	DCA1		**Social Dimension**	DCA1
**1**	Distrito Nacional	0.000	1	Distrito Nacional	0.352
**2**	Santo Domingo	0.173	2	Santo Domingo	0.030
**3**	Montecristi	0.553	3	Montecristi	0.000
**4**	Puerto Plata	0.499	4	Puerto Plata	0.046
**5**	Espaillat	0.325	5	Espaillat	0.052
**6**	María Trinidad Sánchez	0.419	6	María Trinidad Sánchez	0.142
**7**	Samaná	0.582	7	Samaná	0.015
**8**	Hato Mayor	0.580	8	Hato Mayor	0.076
**9**	El Seibo	0.614	9	El Seibo	0.001
**10**	La Altagracia	0.571	10	La Altagracia	0.059
**11**	La Romana	0.523	11	La Romana	0.103
**12**	San Pedro de Macorís	0.506	12	San Pedro de Macorís	0.059
**13**	San Cristóbal	0.555	13	San Cristóbal	0.028
**14**	Peravia	0.609	14	Peravia	0.104
**15**	Azua	0.558	15	Azua	0.024
**16**	Barahona	0.465	16	Barahona	0.156
**17**	Pedernales	0.720	17	Pedernales	0.022

Table S 3.

Detrended Correspondence Analysis (DCA) Results per variable. 1st Axis Scaled – (DCA1 - scaled)

**Ecological Dimension**		**Economic Dimension**		**Productive Dimension**		**Cultural Dimension**		**Social Dimension**	
**var**	**DCA1_scaled**	**var**	**DCA1_scaled**	**var**	**DCA1_scaled**	**var**	**DCA1_scaled**	**var**	**DCA1_scaled**
montane_very_humid_forest_pct	100.00	foreign_air_arrivals_num	100.00	controlled_cultivation_area_m2	100.00	female_university_students_num	100.00	population_density_km2	100.00
endemic_species_num	96.07	private_construction_investment_usd	83.49	irrigation_channels_length_km	74.74	women_local_government_num	99.93	university_enrollment_num	98.05
lower_montane_very_humid_forest_pct	96.03	formal_msme_initiatives_num	75.13	agricultural_producers_num	71.28	cultural_manifestations_num	99.40	private_universities_num	86.36
trans_very_humid_to_lower_montane_rainforest_pct	94.72	tertiary_sector_employment_pct	65.33	livestock_producers_num	69.70	traditional_dances_conserved_num	91.68	universities_total_num	83.34
lower_montane_humid_forest_pct	94.71	economically_active_population_pct	63.31	subsistence_crops_area_pct	67.55	folkloric_manifestations_num	89.78	public_hospitals_num	71.22
subtropical_thorny_forest_pct	92.02	mining_concessions_num	61.14	irrigated_cultivation_area_pct	66.88	traditional_handicrafts_conserved_num	84.69	households_fixed_telephony_pct	70.81
trans_humid_to_lower_montane_dry_forest_pct	90.24	mineral_production_tons	49.18	paved_roads_urban_pct	63.28	caves_num	82.68	province_population_num	68.99
lakes_lagoons_pct	86.69	free_zone_parks_num	46.47	renewable_energy_production_MWh_year	62.12	high_school_enrollment_num	52.24	physicians_per_10000_inhabitants	67.59
subtropical_dry_forest_pct	64.87	free_zone_companies_num	38.63	pasture_area_pct	56.06	public_libraries_num	41.15	technical_higher_education_enrollment_num	61.89

altitudinal_range_masl	60.95	free_zone_employees_num	36.25	public_transport_use_pct	54.04	museums_cultural_centers_num	34.02	primary_health_centers_public_num	57.78
agricultural_drought_threat_index	53.14	national_exports_free_zones_usd_thousands	33.59	households_fixed_telephone_pct	52.96	university_educated_population_num	18.85	housing_units_num	53.59
life_zones_num	52.52	foreign_maritime_arrivals_num	30.18	wild_species_used_crafts_num	52.18	technical_higher_education_enrollment_num	0	households_paved_access_roads_pct	50.97
trans_dry_to_humid_forest_km2	51.64	cumulative_free_zone_investment_usd	16.24	internet_access_pct	51.83	university_enrollment_num	0	urban_population_pct	49.19
protected_areas_pct	47.18	national_exports_free_zones_kg	13.07	housing_units_num	51.38			human_development_index	49.05
forest_cover_pct	46.20	international_maritime_cargo_mt_year	0.00	commercial_seaports_num	50.95			access_drinking_water_home_pct	48.84
area_km2	44.59			artisanal_landing_ports_num	49.03			public_university_enrollment_pct	46.72
water_use_pressure_pct	43.34			fishermen_num	46.95			access_garbage_collection_pct	43.27
hydrographic_basins_km2	42.31			small_fishing_boats_num	45.67			schools_public_private_num	42.64
forest_types_num	40.97			gross_energy_generation_GWh	43.09			neighborhoods_num	41.50
turtle_nesting_sites_num	37.05			sugarcane_plantations_area_pct	37.83			private_universities_pct	41.22
migratory_species_refuge_num	33.16			airports_num	19.76			health_index	40.08
coastline_length_km	32.74			protected_areas_visitors_num_year	19.45			access_electricity_pct	39.95

forest_fire_threat_index	31.37	hotels_num	18.48	population_over_65_pct	39.74
landslide_threat_index	28.87	tourist_ports_num	15.60	registered_mobile_users_pct	39.00
trans_very_humid_to_rainforest_pct	25.51	hotel_bed_density_beds_km2	2.80	average_population_age_under_34	37.59
beaches_dunes_num	25.00	tourism_beds_num	0.33	average_household_size_num	36.32
total_threats_num	24.49	tourism_water_demand_m3_year	0.00	infant_mortality_rate_per_100000	35.80
protected_areas_num	22.50			immigration_rate_pct	34.81
physical_hazards_num	22.27			population_over_100000_num	34.31
subtropical_rainforest_pct	21.87			households_exposed_natural_disasters_pct	33.05
invasive_species_num	21.21			unemployment_rate_pct	31.23
mean_annual_max_temperature_c	20.95			adolescent_pregnancy_rate_pct	30.93
mean_annual_temperature_c	20.83			births_mothers_under_20_pct	30.87
urban_flood_threat_index	20.50			households_general_poverty_pct	29.24
mean_annual_min_temperature_c	20.45			population_under_10000_num	25.91
water_scarcity_threat_index	20.06			municipal_districts_num	23.23

coastal_surge_threat_index	19.55	municipalities_num	22.75
estuaries_num	19.55	households_extreme_multidimensional_poverty_pct	22.42
river_flood_threat_index	19.29	illiteracy_rate_pct	22.23
seismic_threat_index	17.68	population_10000_50000_num	21.46
hurricane_threat_index	16.69	public_landfills_num	20.74
environmental_vulnerability_index	16.65	households_without_sanitation_pct	19.30
trans_humid_to_very_humid_forest_pct	16.48	charcoal_firewood_cooking_use_pct	17.47
subtropical_humid_forest_pct	9.20	population_50000_100000_num	0.00
subtropical_very_humid_forest_pct	7.39		
annual_rainfall_mm	5.94		
productive_soils_I_VI_pct	5.11		
trans_humid_to_dry_forest_pct	0.00		

Table S 1.

Detrended Correspondence Analysis (DCA). Results per variable. 1st Axis Scaled – (DCA1 - scaled) - Ecological Dimension

var	DCA1_scaled	var	DCA1_scaled
montane_very_humid_forest_pct	100.00	trans_very_humid_to_rainforest_pct	25.51
endemic_species_num	96.07	beaches_dunes_num	25.00
lower_montane_very_humid_forest_pct	96.03	total_threats_num	24.49
trans_very_humid_to_lower_montane_rainforest_pct	94.72	protected_areas_num	22.50
lower_montane_humid_forest_pct	94.71	physical_hazards_num	22.27
subtropical_thorny_forest_pct	92.02	subtropical_rainforest_pct	21.87
trans_humid_to_lower_montane_dry_forest_pct	90.24	invasive_species_num	21.21
lakes_lagoons_pct	86.69	mean_annual_max_temperature_c	20.95
subtropical_dry_forest_pct	64.87	mean_annual_temperature_c	20.83
altitudinal_range_masl	60.95	urban_flood_threat_index	20.50
agricultural_drought_threat_index	53.14	mean_annual_min_temperature_c	20.45
life_zones_num	52.52	water_scarcity_threat_index	20.06
trans_dry_to_humid_forest_km2	51.64	coastal_surge_threat_index	19.55
protected_areas_pct	47.18	estuaries_num	19.55
forest_cover_pct	46.20	river_flood_threat_index	19.29
area_km2	44.59	seismic_threat_index	17.68
water_use_pressure_pct	43.34	hurricane_threat_index	16.69
hydrographic_basins_km2	42.31	environmental_vulnerability_index	16.65
forest_types_num	40.97	trans_humid_to_very_humid_forest_pct	16.48
turtle_nesting_sites_num	37.05	subtropical_humid_forest_pct	9.20
migratory_species_refuge_num	33.16	subtropical_very_humid_forest_pct	7.39
coastline_length_km	32.74	annual_rainfall_mm	5.94
forest_fire_threat_index	31.37	productive_soils_I_VI_pct	5.11
landslide_threat_index	28.87	trans_humid_to_dry_forest_pct	0.00

Table S 5

Detrended Correspondence Analysis (DCA). Results per variable. 1st Axis Scaled – (DCA1 - scaled) – Productive Dimension

var	DCA1_scaled	var	DCA1_scaled
controlled_cultivation_area_m2	100	commercial_seaports_num	50.95
irrigation_channels_length_km	74.74	artisanal_landing_ports_num	49.03
agricultural_producers_num	71.28	fishermen_num	46.95
livestock_producers_num	69.70	small_fishing_boats_num	45.67
subsistence_crops_area_pct	67.55	gross_energy_generation_GWh	43.09
irrigated_cultivation_area_pct	66.88	sugarcane_plantations_area_pct	37.83
paved_roads_urban_pct	63.28	airports_num	19.76
renewable_energy_production_MWh_year	62.12	protected_areas_visitors_num_year	19.45
pasture_area_pct	56.06	hotels_num	18.48
public_transport_use_pct	54.04	tourist_ports_num	15.60
households_fixed_telephone_pct	52.96	hotel_bed_density_beds_km2	2.80
wild_species_used_crafts_num	52.18	tourism_beds_num	0.33
internet_access_pct	51.83	tourism_water_demand_m3_year	0.00
housing_units_num	51.38		

Table S 6

Detrended Correspondence Analysis (DCA). Results per variable. 1st Axis Scaled – (DCA1 - scaled) - Economic Dimension

var	DCA1_scaled
foreign_air_arrivals_num	100
private_construction_investment_usd	83.49
formal_msme_initiatives_num	75.13
tertiary_sector_employment_pct	65.32
economically_active_population_pct	63.31
mining_concessions_num	61.13
mineral_production_tons	49.18
free_zone_parks_num	46.46
free_zone_companies_num	38.62
free_zone_employees_num	36.25
national_exports_free_zones_usd_thousands	33.58
foreign_maritime_arrivals_num	30.17
cumulative_free_zone_investment_usd	16.24
national_exports_free_zones_kg	13.07
international_maritime_cargo_mt_year	0

Table S 2

Detrended Correspondence Analysis (DCA). Results per variable. 1st Axis Scaled – (DCA1 - scaled) - Cultural Dimension

var	DCA1_scaled
female_university_students_num	100
women_local_government_num	99.93
cultural_manifestations_num	99.40
traditional_dances_conserved_num	91.67
folkloric_manifestations_num	89.78
traditional_handicrafts_conserved_num	84.69
caves_num	82.67
high_school_enrollment_num	52.23
public_libraries_num	41.14
museums_cultural_centers_num	34.01
university_educated_population_num	18.84
technical_higher_education_enrollment_num	0
university_enrollment_num	0

Table S 3

Detrended Correspondence Analysis (DCA). Results per variable. 1st Axis Scaled – (DCA1 - scaled) - Social Dimension

var	DCA1_scaled	var	DCA1_scaled
population_density_km2	100	population_over_65_pct	39.74
university_enrollment_num	98.05	registered_mobile_users_pct	39.00
private_universities_num	86.36	average_population_age_under_34	37.59
universities_total_num	83.34	average_household_size_num	36.32
public_hospitals_num	71.22	infant_mortality_rate_per_100000	35.80
households_fixed_telephony_pct	70.81	immigration_rate_pct	34.81
province_population_num	68.99	population_over_100000_num	34.31
physicians_per_10000_inhabitants	67.59	households_exposed_natural_disasters_pct	33.05
technical_higher_education_enrollment_num	61.89	unemployment_rate_pct	31.23
primary_health_centers_public_num	57.78	adolescent_pregnancy_rate_pct	30.93
housing_units_num	53.59	births_mothers_under_20_pct	30.87
households_paved_access_roads_pct	50.97	households_general_poverty_pct	29.24
urban_population_pct	49.19	population_under_10000_num	25.91
human_development_index	49.05	municipal_districts_num	23.23
access_drinking_water_home_pct	48.84	municipalities_num	22.75
public_university_enrollment_pct	46.72	households_extreme_multidimensional_poverty _pct	22.42
access_garbage_collection_pct	43.27	illiteracy_rate_pct	22.23
schools_public_private_num	42.64	population_10000_50000_num	21.46
neighborhoods_num	41.50	public_landfills_num	20.74
private_universities_pct	41.22	households_without_sanitation_pct	19.30
health_index	40.08	charcoal_firewood_cooking_use_pct	17.47
access_electricity_pct	39.95	population_50000_100000_num	0.00
